# Explicit Not Implicit Preferences Predict Conservation Intentions for Endangered Species and Biomes

**DOI:** 10.1371/journal.pone.0170973

**Published:** 2017-01-30

**Authors:** Alejandra Echeverri, Megan M. Callahan, Kai M. A. Chan, Terre Satterfield, Jiaying Zhao

**Affiliations:** 1 Institute for Resources, Environment and Sustainability, University of British Columbia, Vancouver, British Columbia, Canada; 2 Department of Psychology, University of British Columbia, Vancouver, British Columbia, Canada; University of Brighton, UNITED KINGDOM

## Abstract

Conservation of biodiversity is determined in part by human preferences. Preferences relevant to conservation have been examined largely via explicit measures (e.g., a self-reported degree of liking), with implicit measures (e.g., preconscious, automatic evaluations) receiving relatively less attention. This is the case despite psychological evidence from other contexts that implicit preferences are more informative of behavior. Thus, the type of measure that predicts conservation intentions for biodiversity is unknown. We conducted three studies to examine conservation intentions in light of people’s explicit and implicit preferences toward four endangered species (sea otter, American badger, caribou, yellow-breasted chat) and four biomes (forest, ocean, grassland, tundra). In Study 1 (*n* = 55), we found that people implicitly preferred caribou most, but explicitly preferred sea otter most, with a significant multiple regression where participants’ explicit preferences dictated their stated intended donations for conservation of each species. In Study 2 (*n* = 57) we found that people implicitly and explicitly preferred forest and ocean over grassland and tundra. Explicit rather than implicit preferences predicted the intended donation for conservation of the ocean biome. Study 3 involved a broader online sample of participants (*n* = 463) and also found that explicit preferences dictated the intended donations for conservation of biomes and species. Our findings reveal discrepancies between implicit and explicit preferences toward species, but not toward biomes. Importantly, the results demonstrate that explicit rather than implicit preferences predict conservation intentions for biodiversity. The current findings have several implications for conservation and the communication of biodiversity initiatives.

## Introduction

Human attitudes can determine actions toward biodiversity conservation, and are informed by people’s explicit preferences, values, emotions, and unconscious motives [1,2]. As such, understanding human preferences and intended actions toward biodiversity can contribute to overall conservation goals. The current study aims to better understand how human motivations impact biodiversity conservation. Specifically, we examine the relationship between preferences for endangered species and biomes and the intended donations to conservation.

Implicit versus explicit preferences regarding important social norms have been key to understanding the difference between what people state as their belief or as important, versus positions revealed through the elicitation of less conscious preferences. Here we use implicit preferences as do psychologists wherein a person’s judgments are said to be based on preconscious automatic evaluations conducted without intention [3–5]. *Implicit* preferences can be measured through the use of the Implicit Association Test (IAT), developed by Greenwald et al. (1998), which measures the strength of association between a particular subject and its positive and negative attributes. In contrast, *explicit* preferences represent conscious judgments that can be assessed through self-reported measures (e.g., surveys). A well-established finding in social psychology shows that most people explicitly express no preference among different human faces of varying races or ethnicities due to social norms. For example, Axt et al. (2014) surveyed almost 98,000 people about their preferences toward different racial groups and did not find statistically significant differences across the ratings of explicit preferences for participants who were White, Asian or Hispanic *p*>0.05. They only found a significant difference for how Black participants explicitly rated the third and fourth highest rated racial group *p* = 0, but not the other groups [6]. However, people may nonetheless reveal implicit preferences by exhibiting faster response times when associating positive words with their own self-identified social category [6]. Although we recognize that “race” is and should be a contested term in the social sciences (as efforts to distinguish biophysical features of racial groups have largely failed), the construct “race” is nonetheless widely meaningful in public life and remains an important social basis through which humans construct their identities. Implicit preferences have also been shown to guide behavior more strongly than explicit preferences, due to people’s tendency to conceal their explicit preferences in order to conform to social norms [7].

People’s positive associations with species and biomes can inform social norms that may lead to their protection. For example, lemurs are protected because they are thought to embody Malagasy ancestors [8], and forests are protected due to local beliefs [9]. Similarly, negative associations with a species can undermine their conservation, as in the case of the aye-aye lemur that is killed because of its perceived association with human deaths [10]. Implicit attitudes are likely to affect human-wildlife interactions, and human perceptions of wildlife [4]. However, in the context of conservation, few studies have compared implicit (through the use of the IAT) and explicit (through self-reported measures) preferences in relation to biodiversity [4]. The IAT has been used to measure people’s connectedness with nature [11] and people’s fears towards snakes and spiders [12]. To our knowledge, no studies have evaluated implicit preferences towards animals and biomes for conservation purposes. Moreover, little research has evaluated whether implicit preferences toward biodiversity are more predictive of conservation behaviors than explicit preferences. Past studies have found that explicit preferences predict motivations behind conservation actions (e.g., [2,13]). Thus, we believe that this study contributes to the growing field of conservation psychology by conducting experiments that measure implicit preferences, as well as explicit preferences, and furthermore attempts to link them to conservation-based behavioral intentions.

### Preferences for Species and Biomes and Influencing Factors

Many researchers have studied people’s preferences for species and biomes but have primarily examined explicit measures [4]. Past studies have shown that explicit preferences correlate with sociocultural factors (e.g., familiarity, [14]), evolutionary factors (e.g., the cute response, [15]), and specific traits of the species or biome (e.g., color, [16]). For example, familiarity (e.g., childhood experiences) has been shown to have a large effect on people’s attitudes toward animals [17], and is positively correlated with preference for biomes [18]. Evolutionary factors may drive preferences towards species and biomes [19,20]. For example, the “cute response” hypothesis predicts that humans are attracted to neotenic features in both humans and animals, such as a large forehead and eyes [15,21]. As with species, preferences for biomes are likely to be based on evolutionary factors [22]. For example, the savanna hypothesis predicts that humans have an innate preference for savanna-like biomes due to their evolutionary history of developing on the savanna biomes of Africa [18]. Likewise, the forest hypothesis predicts that preferences for forests are driven by the possibility that humans may have evolved within dense forests [23–25]. The grassland-woodland hypothesis predicts that humans evolved in a combination of the two biomes, which suggests that humans prefer these biomes [26]. Empirical studies have compared preferences for natural biomes with built biomes [27], but no studies have examined implicit preferences across different natural biomes. Finally, specific traits of species and biomes can also influence preferences. A species’ physical appearance, behavioral traits, and ecological characteristics can shape preferences [16]. Animals phylogenetically closer to humans and those with similar appearances and actions are generally more preferred [14,28]. Larger animals are often preferred over smaller animals, and mammals are the most preferred of all taxonomic groups [29,30]. For biomes, larger canopied trees with low branches are often preferred [31], and the presence of water features also increases preference [32].

### Study Goals

Our goal was to investigate implicit and explicit preferences toward endangered species and biomes. Furthermore, we wanted to determine how these preferences drive behavioral intentions to donate to conservation. Here we use intended donation as the maximum amount of money that people are willing to donate to help conserve a species or a biome. While many behaviors could have been examined (e.g., petition signing, volunteering), intended donation represents one of the most targeted behaviors by conservation organizations [33]. A few studies have examined the willingness to pay for or donate to larger biomes, such as forest and grassland (e.g., [34,35]). However, no studies have compared intended donations for biome conservation across a number of different biomes. Similarly, much work has examined preferences for charismatic animals [36,37], but less work has been done on charismatic biomes. Although the charismatic extent of both flora and fauna have been studied [35,37,38], less is known about what constitutes a charismatic biome as a whole and people’s preferences across biomes.

Three specific objectives thus guided our work: to (1) investigate implicit and explicit preferences for both animal species and biomes (including charismatic endangered species and biomes), (2) evaluate whether implicit or explicit preferences determine people’s intended donations, and (3) qualitatively understand what factors inform perceptions and preferences of species and biomes.

## Methods

This paper addresses findings from three studies that were approved by the University of British Columbia (UBC) Behavioral Research Ethics Board (ethics certificate number H13-02679). We collected data between January and March 2015. The first two studies examined implicit and explicit preferences for endangered species (study 1, *n* = 55) and biomes (study 2, *n* = 57), using the Multi-Category Implicit Association Test (MC-IAT) [6]. We measured implicit preferences using the aforementioned test, alongside a survey to assess explicit preferences including measures of familiarity, perceived levels of endangerment, and intended donations for conserving different species and biomes. Participants also completed a word association task for each species and biome; word associations are credited for revealing images and associations, negative and positive affect, without the full burden of discursive language [39,40]. The third study validated explicit preferences for species and biomes, using a broader sample of participants (*n* = 463) who were recruited via Amazon Mechanical Turk (Mturk). In the analyses, we examined implicit measures (i.e., D scores, [41]) and explicit responses (i.e., preference, familiarity, perceived endangerment or threat) as predictors (independent variables) of the intended donations (the dependent variable).

### Study Tasks and Sampling

#### Study 1

55 undergraduate students at UBC (37 female, 16 male, 2 other; mean age(SD) = 20.9(3.4)) participated in the study in exchange for course credit. Participants were recruited through the human subject pool (HSP) in the Department of Psychology. They were asked to partake in a study entitled “Environmental Perception.” The study consisted of two parts and lasted one hour. After signing a consent form and receiving an instruction sheet, participants first completed a 4-category species MC-IAT and then filled out a survey that measured their explicit preferences toward the species. The four species were: sea otter (*Enhydra lutris*), caribou (*Rangifer tarandus*), American badger (*Taxidea taxus*), and yellow-breasted chat (*Icteria virens*). The species were selected from a pool of 18 species at risk in B.C. in a pilot study (*n* = 134, not described in this paper). We chose the four species that had similar rankings in terms of familiarity and explicit preference and avoided species with high popularity among our pilot study population (e.g., blue whale, spotted owl) to avoid overly strong emotional responses.

The MC-IAT, a variant of the Brief IAT [41], measured the association strength between species and positive attributes and provided a measure of implicit preference. The test contained 13 blocks (the first block was the practice block). Each block had 16 trials presenting 16 different stimuli and each trial was either a word or an image. Participants were asked to press the “I” key for any “good words” (love, pleasant, great, wonderful), or for images of one focal species (e.g., otter), and to press the “E” key for any “bad words” (hate, unpleasant, awful, terrible), or for images of the non-focal species (e.g., caribou). Each block contained a different combination of focal and non-focal species. For example, there were three blocks in which participants pressed the “I” key for sea otter: in one block sea otters and good words were presented against caribou and bad words, in another block they were presented against American badger and bad words, and in another block against yellow-breasted chat and bad words. Importantly, each species was paired with good words for the same number of times as they were paired with bad words. Participants first completed the practice block and then the 12 blocks in a random order. We used MATLAB to run the task and collect participants’ responses.

D scores were calculated from the MC-IAT [42]. A D score was computed for each contrast by subtracting the mean response time (RT) for one block (e.g., sea otters with good words, caribou with bad words), from the mean RT for the other block (e.g., caribou with good words, sea otters with bad words) and then dividing by the SD of the RTs across both blocks. There were six D scores representing the contrast between each pair of species (sea otter vs. caribou, sea otter vs. yellow-breasted chat, sea otter vs. American badger, caribou vs. yellow-breasted chat, caribou vs. American badger, yellow-breasted chat vs. American badger). To calculate each D score, only correct trials were used and all trials with RTs larger than three SDs away from the mean of participants’ RT were removed. We calculated the D score for each participant for each species in order to evaluate if the participant had implicit preferences for certain species over others. All analyses were conducted in R. The four aggregate scores were interdependent, because knowing three scores directly implied the fourth, and the mean of the four scores was necessarily zero [6]. Positive D scores indicate that the species were more preferred than the average score of the four species, and negative D scores indicate that the species were less preferred than the average score of the four species [6].

In addition to the MC-IAT, participants completed a survey with five blocks of questions. The first block was a word association task where participants were asked to write three words that came to mind when they thought of each species. The second block assessed intended donation by asking participants to type in the amount of money they were willing to donate to conserve each species. The third block assessed explicit preference and perceived endangerment by asking participants to rank the species (from 1 = most favorite to 4 = least favorite) and also rank them on how endangered they thought each species was. The fourth block assessed participants’ explicit preferences by asking participants to rate how much they liked each species (from 0 = not at all to 10 = extremely), and also their familiarity with each species. The fifth block asked participants for their demographic information. We used Qualtrics to run the survey and collect participants’ responses.

#### Study 2

57 undergraduate students from UBC participated in the study (44 female, 13 male, mean age(SD) = 19.8(2.0)). This study was identical to study 1, except with four biomes (forest, ocean, grassland, tundra) instead of four species. Participants completed the MC-IAT with the biomes followed by a survey that measured their explicit preferences and familiarity with each biome, and their intended donation to conserve each biome. In addition, they completed the word association task with each biome, ranked the biomes in terms of how threatened they thought each biome was, and answered demographic questions.

#### Study 3

To generalize our findings from the explicit preferences to a broader population, we recruited 463 participants (208 female, 253 male, 2 other) via Amazon Mechanical Turk (Mturk), an online crowdsourcing platform that enables researchers to conduct studies with a larger, worldwide participant sample. Participants each received US$0.50 in exchange for their participation. Their ages ranged from 18 to 69 years old (mean age(SD) = 35.2(11.5)). The majority of participants were located in the United States (76%), followed by India (21%). Participants completed a survey with 10 blocks of questions. Blocks 1–5 were the same questions about the four species as in the survey in study 1, and blocks 6–10 were the same questions about the four biomes as in the survey in study 2. The MC-IAT was not run in this study because of the need for a controlled laboratory environment to capture accurate response times and ensure participants’ full attention. Response times in online web-based studies are elusive due to uncontrolled conditions including environment, Internet speeds, and others.

### Justification of Sampling Methods

Online and student sampling in experiments is a widely accepted practice in psychology. While we acknowledge that using a student sample has limitations because they may not fully reflect the preferences of broader populations. College-age individuals can often be influenced because emerging adults are constantly exploring self-identities [43] and thus are of special interest to conservation and advocacy organizations. Amazon Mechanical Turk (Mturk) allows for a larger, relatively more global population with a wide-range of income. Thus, participants on Mturk also represent individuals who would donate to conservation. Based on previous MC-IAT studies in the lab, we conducted a power analysis and determined that a sample size of 50 would be sufficient for studies 1 and 2. In the third study, we aimed to validate the explicit preference portion of our study, using the larger and more diverse sample.

### Data Analysis

#### Quantitative analysis

We used paired t-tests to test the differences between the D scores, to evaluate people’s implicit preferences of the species and biomes in studies 1 and 2. For the explicit preferences, we used one-way ANOVAs to test the differences in ratings on preferences, familiarity, perceived endangerment, and intended donation across the four species and biomes in all three studies. We used post-hoc Tukey’s HSD tests to elucidate further pairwise differences. In addition, for all participants we also ran correlation tests between D scores and ratings of preferences for every species and biome. Additionally, for all studies we ran multiple regressions to examine which factors predicted intended donation. The predictors were D scores (as a measure of implicit preferences), explicit preferences, familiarity, and perceived endangerment (in study 3 we excluded the D scores as a predictor since it was not measured). We only used data from participants who intended to donate between $0–300 for studies 1 and 2, and between $0–10,000 for study 3. This excluded extreme responses that we interpreted as protest votes (e.g., $1 trillion). The limit for intended donations was lower for studies 1 and 2, than for study 3 because all participants in the first two studies were students. In study 3 only 10% of the participants were students, and the majority were employed with higher income levels. All statistical analyses were conducted in R.

#### Qualitative analysis

We used non-hierarchical axial coding to code participants’ responses in the word association task. First, based on the words described by the participants, we created 12 labels (Table 1). Second, we assigned each word to one of the labels and then counted the frequency of words within each label. Next, we conducted a second round of coding using only two broad categories (positive associations and negative associations) to match the results from the MC-IAT. Finally, we conducted chi-squared tests to evaluate the statistical differences between the frequencies of positive and negative associations for each species and each biome.

**Table 1:** Labels used in the coding analysis of the word association task for studies 1, 2 and 3.

Label	Meaning	Examples
Descriptors	Words used to describe the essence of the animal or biome or its main appearance	Animal, Mammal, Big, White, Cold, Windy, Foggy
Behavioral traits	Words used to describe common behaviors of the animal	Active, Calm, Cautious, Eager
Environment	Words used to describe the ecosystem where the animal lives	Grassland, Kelp, Ocean, Water, Winter, Woods
Actions	Words used to describe a common activity that the animal does	Swim, Sing, Chirp, Fly
Part of body	Words that refer to prominent body parts of the animal	Antlers, Whiskers, Claws, Wings
Equivalents	Words that refer to a different but similar animal or biome than the evaluated animal or biome	Moose, Raccoon, Skunk, Plains, Savannah, Desert
Commercial/utilitarian	Words that refer to a commercial product derived from the animal or that refer to branding	Beer, Coin, Youtube video, sports team mascot
Recreation	Words that refer to a recreational activity involving the animal or its ecosystem	Aquarium, Wetsuit, Surf
Otherworldly	Words that refer to fantasy	Unicorn, Magic, Mythical, Pixies, Mermaids
Unknown	Words that indicate lack of knowledge or lack of familiarity with the animal	Never seen, Don't know
Positive associations	Words that have positive connotations when describing the animal or biome	Beautiful, Majestic, Pretty, Cute, Peaceful, Happy
Negative associations	Words that have negative connotations when describing the animal or biome	Annoying, Awful, Bad, Hate, Unattractive, Violent, Danger
Colors	Words that indicate the colors associated with the biomes	Brown, Blue, Yellow, Very Green
Comprising	Words that refer to flora, fauna, and other natural features that are found within the biome	Water, Sky, Shark, Trees
Activities	Words that represent an activity that is undertaken within the biome	Walk, Swimming, Surfing, Grazing
Geography	Words referring to the overall location of a biome	Hawaii, Canada, Africa

## Results

### Study 1

**Fig 1:**
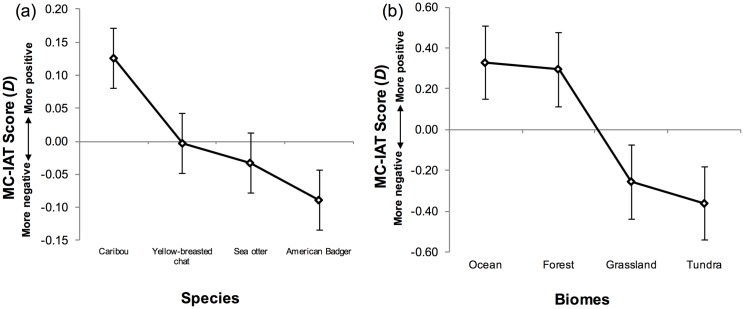
Implicit preferences for species and biomes. D scores as a measure of implicit preference from the Multi-Category Implicit Association Test (MC-IAT) for (a) the four species in study 1 and (b) the four biomes in study 2. Each diamond represents the average D score with standard error bars.

**Fig 2:**
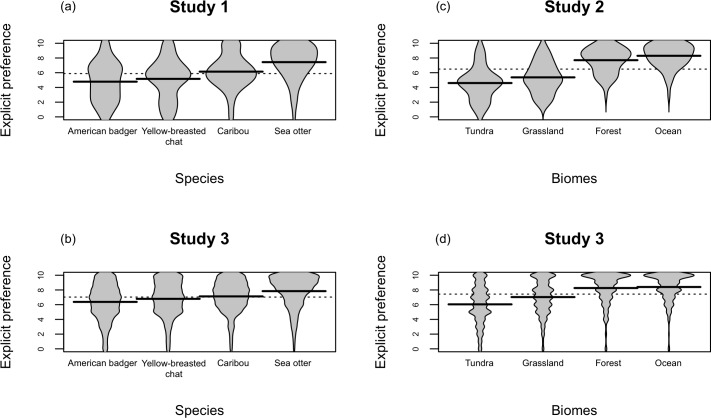
Explicit preferences for species and biomes. Beanplots showing the results for explicit preference ratings of (a) species among lab participants (*n* = 55) in study 1, (b) species among Mturk participants (*n* = 463) in study 3, (c) biomes among lab participants (*n* = 57) in study 2, (d) biomes among Mturk participants (*n* = 463) in study 3. Each bean (i.e., individual polygon for each species or biome) consists of a density trace showing the distribution of the ratings that is mirrored to form a polygon shape. Solid black lines represent the mean rating for each polygon, and the dotted line indicates the grand mean.

D scores from the MC-IAT, which are a measure of implicit preferences, are shown in Fig 1(a). Paired *t*-tests revealed the only statistically significant difference: caribou was implicitly more preferred than American badger (*t*(54) = 2.36, *p* = 0.02, *d* = 0.38). For explicit preferences, participants’ ratings were shown as beanplots [44] in Fig 2(a). Each beanplot displays the distribution of the rating responses for each species, with the sea otter being most preferred (*p*<0.005). A one-way ANOVA with Tukey’s HSD post-hoc comparisons showed there was a significant difference among all four species (*F*(3,216) = 11.90, *p*<0.001, η_*p*_^2^ = 0.014). The results also demonstrated that implicit preferences of the four species did not align with explicit preferences suggesting that the order of preferences were different between the two measures. Specifically, the pooled results showed that people implicitly preferred caribou but explicitly preferred sea otter. In addition, when pairing D scores and explicit ratings for each participant, the correlations were not significant (*p*>0.05) for every species (Table 2).

**Table 2:** Correlation results for implicit and explicit attitudes between explicit and implicit attitudes for studies 1 and 2.

Study	Species/Biome	t	Correlation estimate	df	p value
1	Caribou	0.938	0.128	53	0.353
	American badger	1.584	0.213	53	0.119
	Sea otter	-0.594	-0.081	53	0.550
	Yellow-breasted chat	1.279	0.173	53	0.207
2	Forest	0.028	0.004	55	0.978
	Ocean	1.882	0.246	55	0.065
	Grassland	-0.848	-0.113	55	0.400
	Tundra	-1.493	-0.197	55	0.141

There were positive correlations across respondents between explicit preference and familiarity for caribou (*r*(53) = 0.30, *p* = 0.02), American badger (*r*(53) = 0.35, *p* = 0.008), sea otter (*r*(53) = 0.48, *p*<0.001), and yellow-breasted chat (*r*(53) = 0.31, *p* = 0.02). This suggests that the more familiar people were with the species, the more they explicitly liked the species. There was a significant difference in familiarity (*F*(3,216) = 34.49, *p*<0.001, η_*p*_^2^ = 0.32), where sea otter was rated as the most familiar (Tukey’s HSD *p*<0.05). There was a significant correlation between implicit preference (D scores) and familiarity for caribou (*r*(53) = -0.26, *p*<0.05), but not for the other species (*p*>0.05).

The vast majority (90%) of participants (*n* = 52) reported that they intended to donate between $0-$300 for species conservation. For these respondents, there were positive correlations between their intended donation and their explicit preferences for each species: caribou (*r*(50) = 0.39, *p* = 0.004), American badger (*r*(51) = 0.41, *p* = 0.002), sea otter (*r*(51) = 0.31 *p* = 0.02), and yellow-breasted chat (*r*(51) = 0.46, *p*<0.001). Multiple regressions with all predictors of the intended donation (normalized using box cox transformation) were significant for each species: caribou (*Adj-R*^*2*^ = 0.18, *F*(4,47) = 3.79, *p* = 0.009), American Badger (*Adj-R*^*2*^ = 0.36, *F*(4,48) = 8.19, *p*<0.001), sea otter (*Adj-R*^*2*^ = 0.35, *F*(4,48) = 8.03, *p*<0.001), and yellow-breasted chat (*Adj-R*^*2*^ = 0.27, *F*(4,48) = 5.85, *p*<0.001). The beta for each predictor is listed in Table 3. For every species, explicit preference was the significant predictor of intended donation. These results suggest that explicit preference, not implicit preference, predicted conservation intentions as measured by intended donation. In addition, for sea otter and American badger the perceived endangerment also significantly predicted intended donation (Table 3).

**Table 3:** Results of multiple regression analyses for intention to donate as dependent variable in studies 1, 2, and 3.

Intention to donate for conservation of	Predictor variable	Estimate	SE	t value	p value
Study 1					
Sea otter	D score	-0.010	0.163	-0.094	0.925
	Explicit preference	0.090	0.029	3.101	<0.01
	Familiarity	0.040	0.020	1.848	0.070
	Perceived endangerment	0.160	0.050	2.730	0.008
Caribou	D score	-0.240	0.220	-1.070	0.286
	Explicit preference	0.150	0.047	3.198	<0.01
	Familiarity	-0.016	0.043	-0.391	0.697
	Perceived endangerment	-0.100	0.090	-1.202	0.235
American badger	D score	0.111	0.170	0.648	0.520
	Explicit preference	0.089	0.020	3.709	<0.001
	Familiarity	0.050	0.030	1.440	0.150
	Perceived endangerment	-0.140	0.050	-2.570	0.013
Yellow-breasted chat	D score	0.060	0.180	0.348	0.730
	Explicit preference	0.127	0.030	4.163	<0.001
	Familiarity	0.050	0.040	1.197	0.237
	Perceived endangerment	0.020	0.006	0.306	0.761
Study 2					
Forest	D score	-0.710	0.610	-1.160	0.255
	Explicit preference	0.299	0.180	1.660	0.109
	Familiarity	-0.010	0.140	-0.130	0.898
	Perceived threat	0.410	0.288	1.440	0.160
Ocean	D score	0.572	0.407	1.406	0.172
	Explicit preference	0.347	0.132	2.616	0.015
	Familiarity	-0.095	0.103	-0.920	0.366
	Perceived threat	-0.276	0.270	-1.010	0.319
Grassland	D score	-0.070	0.327	-0.244	0.808
	Explicit preference	-0.028	0.087	-0.331	0.743
	Familiarity	-0.045	0.082	-0.554	0.583
	Perceived threat	-0.364	0.245	-1.483	0.148
Tundra	D score	-39.114	24.339	-1.607	0.118
	Explicit preference	-3.877	3.990	-0.971	0.339
	Familiarity	7.018	4.313	1.627	0.114
	Perceived threat	-14.188	7.036	-2.017	0.053
Study 3					
Sea otter	Explicit preference	0.016	0.003	4.765	<0.001
	Familiarity	0.005	0.003	1.768	0.078
	Perceived endangerment	-0.012	0.006	-2.070	0.039
Caribou	Explicit preference	0.019	0.003	5.545	<0.001
	Familiarity	0.008	0.003	2.696	0.007
	Perceived endangerment	-0.007	0.006	-1.179	0.239
American badger	Explicit preference	0.016	0.003	5.467	<0.001
	Familiarity	0.007	0.003	2.304	0.022
	Perceived endangerment	0.002	0.007	0.278	0.781
Yellow-breasted chat	Explicit preference	0.006	0.001	5.361	<0.001
	Familiarity	0.003	0.001	4.022	<0.001
	Perceived endangerment	0.001	0.002	0.757	0.449
Forest	Explicit preference	0.091	0.024	3.700	<0.001
	Familiarity	0.040	0.023	1.712	0.088
	Perceived threat	-0.006	0.040	-0.137	0.891
Ocean	Explicit preference	0.014	0.004	3.393	<0.001
	Familiarity	0.004	0.004	1.146	0.252
	Perceived threat	-0.005	0.006	-0.835	0.404
Grassland	Explicit preference	0.031	0.006	4.999	<0.001
	Familiarity	0.008	0.005	1.553	0.121
	Perceived threat	-0.017	0.010	-1.248	0.213
Tundra	Explicit preference	0.017	0.003	5.555	<0.001
	Familiarity	0.004	0.002	1.715	0.087
	Perceived threat	-0.004	0.007	-0.531	0.596

**Fig 3:**
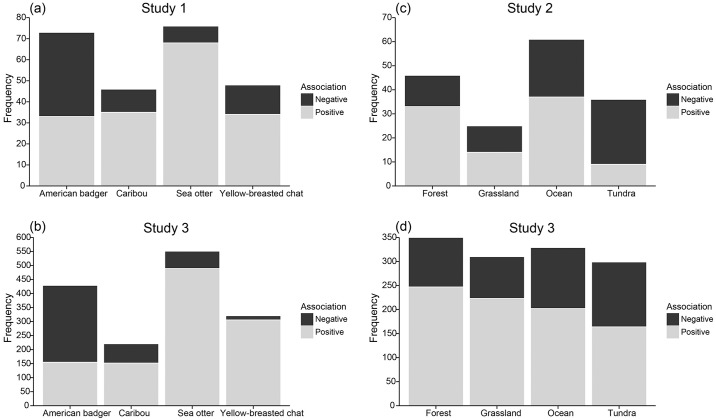
Word association results for species and biomes. Stacked columns bar graphs showing the results of the word association task where participants wrote the words that came to mind when thinking about the species or biomes. The words were coded into positive and negative associations. The frequencies of the words were (a) about species among lab participants (*n* = 55) in study 1, (b) words about species among Mturk participants (*n* = 463) in study 3, (c) about biomes among lab participants (*n* = 57) in study 2, (d) about biomes among Mturk participants (*n* = 463) in study 3.

The word association task showed significant differences between the number of negative and positive words associated with the species (χ^2^(3) = 35.89, *p*<0.001). As shown in Fig 3(a), sea otter had more positive associations than expected by chance, and American badger was the only species with more negative associations than positive ones (*n(*negative) = 40; *n(*positive) = 33) (Fig 3). This qualitative finding was consistent with the ratings of explicit preferences, in that sea otter was the most preferred and American badger was the least preferred.

### Study 2

Study 2 used similar analyses to Study 1 to examine people’s preferences and conservation intentions for biomes. D scores from the MC-IAT, a measure of implicit preference, are shown in Fig 1(b). Paired *t*-tests revealed that people implicitly preferred forest and ocean over grassland and tundra (forest vs. grassland *t*(56) = 4.87, *p*<0.001, *d* = 0.45; forest vs. tundra *t*(56) = 7.39, *p*<0.001, *d* = 0.42; ocean vs. grassland *t*(56) = 4.19, *p*<0.001, *d* = 0.48, ocean vs. tundra *t*(56) = 6.09, *p*<0.001, *d* = 0.45). For explicit preferences, participants’ ratings were shown in Fig 2(c). One-way ANOVAs revealed that there was a significant difference among the four biomes (*F*(3,224) = 28.01, *p*<0.001, η_*p*_^2^ = 0.027), with Tukey’s HSD post-hoc comparisons showing that forest and ocean were more preferred than grassland and tundra (*p*<0.001), but forest and ocean, and grassland and tundra were not different (*p*>0.05). Unlike Study 1, the pooled results showed that implicit and explicit preferences were aligned in terms of their order of preference, in that forest and ocean were preferred over grassland and tundra. However, correlations were also non-significant for every biome (*p*>0.05), indicating that D scores and explicit preferences were not correlated for each participant (Table 2).

There were positive correlations between explicit preference and familiarity for forest (*r*(55) = 0.46, *p*<0.001), ocean (*r*(55) = 0.41, *p*<0.05), grassland (*r*(55) = 0.51, *p*<0.001), and tundra (*r*(55) = 0.68, *p*<0.001). Thus, the more familiar people were with each biome, the more they explicitly liked the biome. There was a significant difference in familiarity (*F*(3,224) = 48.80, *p*<0.001, η_*p*_^2^ = 0.39), with people being more familiar with forest and ocean than with grassland and tundra (Tukey’s HSD *p*<0.001 for all comparisons). There was a significant negative correlation between implicit preference (D scores) and familiarity for forest (*r*(55) = -0.39, *p*<0.05), but no other significant correlations were found for the other biomes (*p*>0.05).

A majority (70.6%) of participants (*n* = 36) reported that they intended to donate between $0 and $300 for biome conservation. Multiple regressions with all predictors of intended donation (normalized using box cox transformation) showed that for ocean, explicit preference predicted intended donation (*Adj-R*^*2*^ = 0.24, *F*(4,26) = 3.36, *p*<0.05). No other regressions were significant (see Table 3).

The word association task showed significant differences between the number of negative and positive words associated with the biomes (χ^2^(3) = 19.11, *p*<0.001). As shown in Fig 3(c), forests and ocean had more positive associations than expected by chance, and tundra had more negative than positive associations (*n(*negative) = 27; *n(*positive) = 9). This finding was consistent with both implicit and explicit preferences, in that forest and ocean were preferred over grassland and tundra.

### Study 3

#### Species results

For Mturk participants, there were significant differences in their explicit preferences among the four species (*F*(3,1848) = 32.92, *p*<0.001, η_*p*_^2^ = 0.05), as shown in Fig 2(b). Tukey’s HSD post-hoc comparisons showed that sea otter was the most preferred among all species (*p*<0.05), followed by caribou and yellow-breasted chat, which were equally liked (*p*>0.05), and American badger was the least preferred (*p*<0.05). The results were consistent with the pattern observed in study 1.

Moreover, there was a positive correlation between familiarity and explicit preference for all species: sea otter (*r*(461) = 0.51, *p*<0.001), caribou (*r*(461) = 0.50, *p*<0.001), yellow-breasted chat (*r*(461) = 0.52, *p*<0.001), and American badger (*r*(461) = 0.46, *p*<0.001). This suggests that the more familiar people were with the species, the more they explicitly liked the species. There were also significant differences in familiarity (*F*(3,1848) = 90.97, *p*<0.001, η_*p*_^2^ = 0.13), with sea otter and caribou being rated as the most familiar with no difference between the two (Tukey’s HSD *p*>0.05). Next was American badger (Tukey’s HSD *p*<0.05), with yellow-breasted chat being the least familiar (Tukey’s HSD *p*<0.05).

A large majority (99.3%) of participants (*n* = 460) reported that they were willing to donate between $0 and $10,000 for species conservation. Even after normalizing the intended donation data with box cox transformations we got slightly skewed data, but after running regressions without transformation and with logarithmic transformation we still obtained the same results. We found a positive correlation between intended donation and explicit preference for caribou (*r*(457) = 0.11, *p*<0.05), but it was not significant for the other species (*p*>0.05). Multiple regressions with all predictors of intended donation (after box cox transformation) were significant for each species: caribou (*Adj-R*^*2*^ = 0.13, *F*(3,455) = 24.25, *p*<0.001), American Badger (*Adj-R*^*2*^ = 0.11, *F*(3,455) = 19.88, *p*<0.001), sea otter (*Adj-R*^*2*^ = 0.09, *F*(3,454) = 17.78, *p*<0.001), and yellow-breasted chat (*Adj-R*^*2*^ = 0.17, *F*(3,455) = 31.51, *p*<0.001). As shown in Table 3, explicit preference significantly predicted intended donation for each species. Similar to the results from study 1, familiarity predicted the intended donation for caribou, American badger, and yellow-breasted chat. For sea otter, perceived endangerment predicted intended donation.

In the word association task, there were significant differences between the number of positive and negative associations recorded among species (χ^2^(3) = 442.05, *p*<0.001). As in study 1 and as shown in Fig 3(b), sea otter and yellow-breasted chat had more positive associations than expected by chance, and American badger was the only species with more negative than positive associations (*n(*negative) = 274; *n(*positive) = 155).

#### Biomes results

As shown in Fig 2(d), Mturk participants showed different explicit preferences for the biomes (*F*(3,1848) = 114.2, *p*<0.001, η_*p*_^2^ = 0.16). Specifically, forest and ocean were the most preferred (with no difference between the two, Tukey’s HSD *p*>0.05), followed by grassland and tundra (Tukey’s HSD *p*<0.05). The same pattern was found for familiarity: participants were more familiar with forest and ocean (with no difference between the two, Tukey’s HSD *p*>0.05), then grassland and then tundra (Tukey’s HSD *p*<0.05). Consistent with the results of study 2, there was a positive correlation between familiarity and explicit preference for every biome: forest (*r*(461) = 0.54, *p*<0.001), ocean (*r*(461) = 0.54, *p*<0.001), grassland (*r*(461) = 0.50, *p* < .001), and tundra (*r*(461) = 0.49, *p*<0.001). This suggests that the more familiar people were with the biome, the more they liked the biome.

A majority (85.7%) of participants (*n* = 397) reported that they intended to donate between $0 and $10,000 for biome conservation. There was a positive correlation between intended donation and explicit preference for grassland (*r*(377) = 0.11, *p*<0.05). Even after normalizing the intended donation variable we got slightly skewed data, but as with the species data, we ran the regressions without transformation and with logarithmic transformation and we still obtained the same results. Multiple regressions with all predictors of intended donation (after using box cox transformation) were significant for each biome: forest (*Adj-R*^*2*^ = 0.06, *F*(3,419) = 10.61, *p*<0.001), ocean (*Adj-R*^*2*^ = 0.05, *F*(3,453) = 8.48, *p*<0.001), grassland (*Adj-R*^*2*^ = 0.09, *F*(3,453) = 16.21, *p*<0.001), and tundra (*Adj-R*^*2*^ = 0.11, *F*(3,454) = 19.31, *p*<0.001). For every biome, explicit preference was the only significant predictor of intended donation (see Table 3). These results provide stronger evidence than those in study 2, suggesting that explicit preferences predicted intended donation for conservation of biomes.

The word association task showed significant differences between the number of negative and positive words associated with the biomes (χ^2^(3) = 26.7, *p*<0.001). As shown in Fig 3(d), forest, ocean, and grassland had more positive associations than expected by chance.

## Discussion

As with other contexts (e.g., racism), we found a discrepancy between implicit and explicit preferences for endangered species, but not for biomes; in contrast to findings from these other contexts, we found that explicit, rather than implicit preferences predicted an individual’s intended donation. In study 1, we found that implicit preferences of the species did not align with explicit preferences. Specifically as a group, people implicitly preferred caribou the most, but explicitly preferred sea otter the most. For every species, explicit preference predicted intended donation and was correlated with familiarity. Thus, explicit preference, not implicit preference, predicted conservation intentions. In study 2 however, we found that implicit and explicit preferences were strongly aligned by order of preference at the group level, and forest and ocean were both preferred over grassland or tundra. Explicit preference again predicted intended donation and was correlated with familiarity, consistent with previous studies [18]. Study 3 replicated the explicit preference portion of both studies with a broader sample of participants.

The three studies have several implications for conservation purposes. First, in most cases conservation efforts and studies often focus on species, but comparatively biomes are poorly understood. Our results add to the literature by suggesting that for biomes, explicit preferences determine intended donation, and these preferences are correlated with familiarity. Second, our results indicate more clearly the factors underpinning intended donations for both species and biomes. Third, by using word association, we elucidate perceptions that may inform explicit preferences (i.e. American badger associated with “mean” and tundra with “barren”). It was necessary to run all three studies in order to make comparisons between species and biomes and the factors that seem to inform conservation intentions.

For species, sea otters were strongly associated with the cute response, validated by the word association task (i.e. “cute” was the most frequent word in study 1 (*n* = 30 = 54.5%) and study 3 (*n* = 168 = 36.4%)). The cute response has been shown to influence explicit preference [15,21]. However, for implicit responses, caribou was the most preferred, likely driven by its large body size. For example authors have found that larger animals are more popular than smaller animals among zoo visitors [30], and that after evaluating preferences for 154 species, the top rated animals were the largest or aesthetically attractive [45]. In fact many of the most frequently used words about caribou in the word association task were “big,” “large,” and “strong.” Heavy media coverage in disputes about pipelines and the fragmentation of caribou habitat may have influenced the observed results [46]. In addition, American badger was least preferred explicitly (in studies 1 and 3). These results may be explained by the qualitative data on the word association task, which showed that it was the only species with more negative than positive associations (Fig 3). Lab participants used words such as angry (*n* = 4 = 7.3%), terrible (*n* = 3 = 5.5%), unpleasant (*n* = 3 = 5.5%), and mean (*n* = 3 = 5.5%) in relation to American badger, while Mturk participants used mean (*n* = 66 = 14.3%), aggressive (*n* = 24 = 5.2%), tough (*n* = 24 = 5.2%), and dangerous (*n* = 19 = 4.1%). Since this is a terrestrial (rather than marine) species, people may relate to it more as a risk or a threat to livestock, pets, and property.

Our results may be interpreted through the affect heuristic proposed by Finucane et al. (2000) [47] and Slovic, et al. (2007), which suggests that relying on positive and negative feelings can predict and explain perceived risks. With regards to stimuli (in this case technological hazards), Slovic et al. (2007) stated that if a person likes an activity they perceive the risk associated with that activity as low, and if they dislike the activity they perceive the risk as high. Our findings regarding the American badger can possibly be understood via the affect heuristic: that is, people explicitly liked the badger the least and perceived it as the most threatening or “risky” among the four species.

For biomes, forest and ocean were more preferred than grassland and tundra because of the greater familiarity of forests and oceans. Tundra was least preferred presumably because it was the only biome with more negative than positive associations among lab participants (study 2) who used words such as barren (*n* = 11 = 18.9%), boring (*n* = 4 = 6.9%), dangerous (*n* = 4 = 6.9%), and death (*n* = 4 = 6.9%) to describe it. This result supports the idea that fear is an important factor in influencing people’s preferences [20], with emotional responses to risky situations (or species) as particularly important and predicted by the affect heuristic [40,48].

We also found that conservation intentions for both species and biomes, as indicated by intended donations, were predicted by explicit preferences, not implicit preferences in all cases (Table 3). One notable finding is that for lab participants, explicit preferences predicted intention to donate for all species (study 1) but only for ocean across biomes (study 2). In contrast, for Mturk participants, explicit preferences predicted intention to donate for all biomes, and only for caribou (study 3).

The current study has important ethical implications. The principle of human equality is widely accepted and moral principles in many contemporary societies state that human races are equal despite racial, gender, mobility, income, or other bases of differentiation [49]. In the IAT studies evaluating social issues, findings are consistent with those principles because explicitly people respond in a conscious way to abide to those moral principles and answer that all races have equal moral standing, but implicitly a difference of attitudes toward racial groups is shown by the IAT. However, when evaluating preferences toward species and biomes, this study showed that participants appeared to feel no need to hide preferences for particular species or biomes. Thus, biospherical egalitarianism—the principle that all species and biomes have equal moral standing [50,51], does not seem to apply as a moral principle guiding conscious judgments toward species and biomes. Schmidtz (1998) argued that speciesism seems to apply more for societal perceptions of species, and that animals are often ranked in a moral scale with some having perceived superiority over others (e.g., if chimpanzees were used in medical trials rather than mice many more people would oppose to such experiments) [50]. In addition, Greenwald, et al. (2009) found that explicit attitudes (self-reported) were better predictors of behaviors in cases when stating preferences was not controversial (i.e. low social desirability) [7]. Thus this idea of speciesism, or in other words a lack of biospherical egalitarianism, is perhaps a fundamental basis for our results because explicit preferences were much better predictors of intended donations than implicit preferences.

Even though we found more robust results for explicit preferences, we should also consider the limitations of our tests of implicit preferences. It is important to note that even though we conducted power analyses to choose our sample sizes for studies 1 and 2 based on studies conducted in our lab, other studies that have used MC-IATs have used extremely large samples (e.g., in Axt, et al. 2014). Perhaps more participants may have been needed to get more reliable estimates of differences in implicit evaluations among species and biomes, but it was notable that we found differences nonetheless. We are aware that the MC-IAT is a noisy measure because a few trials must determine an aggregate score, and thus increasing sample sizes should be a priority for future research using MC-IATs.

The current findings can inform conservation campaigns that address endangered species and biomes by eliciting public support for policies tailored to specific species [52]. Perhaps unsurprisingly, given the focus of conservation marketing on charismatic species, people’s explicit preference toward a species was positively correlated with the intended donation for conserving that species. However, the interesting contribution of this study is that the same principle applies to charismatic biomes. Our study is among the first to show that explicit preference predicts the intended donation for the conservation of biomes and that such preferences are informed by familiarity.

Conservation organizations addressing issues of endangered species and biomes might benefit from considering preferences and the negative versus positive associations. For example, if an organization needs to raise funds for the conservation of the American badger or the tundra, campaigns might focus on portraying them in more positive terms, generating more positive affective attitudes toward them. Increasing familiarity could be accomplished through exposure to positive messaging, which could be found in various spheres (e.g. movies, sports). Additionally, the results may be used for selecting more favored (flagship) species for fundraising purposes. Further research can focus on running the IAT using different words for the associations with biomes and species. It may be useful to design a new IAT with the words that were recorded as common in the word association task in order to test if implicit preferences toward animals and biomes differ when different words are used. Further research might also evaluate how intended donation predicts actual donation behavior.

## Supporting Information

S1 Table Participant demographics for study 1. (PDF)

S2 Table Participant demographics for study 2. (PDF)

S3 Table Participant demographics for study 3. (PDF)

S4 Table Creative commons licenses for the pictures used in the MC-IAT of species and biomes in studies 1, 2 and 3. (PDF)

S1 Fig Instruction page for the MC-IAT for Study 1 and 2. (PDF)

S2 Fig Questionnaire for study 1. (PDF)

S3 Fig Questionnaire for study 2. (PDF)

S4 Fig Questionnaire for study 3. (PDF)
