# Supplementary material for: Explicit Not Implicit Preferences Predict Conservation Intentions for Endangered Species and Biomes
Source: PLoS One. 2017 Jan 30;12(1):e0170973. doi: 10.1371/journal.pone.0170973 (PMC5279788; doi:10.1371/journal.pone.0170973)
Supplement: S1 Table — (PDF) [file pone.0170973.s001.pdf]

S1 Table. Participant demographics for study 1.

Variable	*n*	Percentage
**Ethnicity**		
White or Caucasian	12	21.82%
Black or African-American	1	1.82%
Hispanic or Latino (includes Mexican, Central American and South American)	2	3.64%
Korean	8	14.55%
Chinese	23	41.82%
Filipino	1	1.82%
Middle eastern	1	1.82%
South Asian (from India, Bangladesh, Pakistan, etc)	2	3.64%
Other	1	1.82%
Multiracial	4	7.27%
**Total**	**55**	**100.00%**
**Highest level of education completed**		
High school or equivalent	43	78.18%
College	1	1.82%
Bachelor's degree	9	16.36%
Professional degree (MD, JD, etc)	1	1.82%
Master's degree	1	1.82%
**Total**	**55**	**100.00%**
**Employment (Select more than one)**		
Student	55	
Unemployed	18	
Agriculture, forestry, fishing, hunting	1	
Arts, entertainment, recreation	3	
Education College/University	3	
Education Primary/Secondary	1	
Finance and insurance	1	
Business, marketing, administration	1	
Health Care, social assistance	3	
Scientific or technical services	2	
Transportation	1	
Other	5	
**Religious affiliation**		
Muslim	2	3.64%
Orthodox Church such as Greek or Russian Orthodox Church	1	1.82%
Buddhist	3	5.45%

Variable	*n*	Percentage
Catholic	5	9.09%
Protestant	3	5.45%
Jewish	3	5.45%
Atheist	13	23.64%
Agnostic	9	16.36%
Other	16	29.09%
**Total**	**55**	**100.00%**
**Annual household income**		
Less than US$20.000	15	27.27%
US $20.001-$40.000	8	14.55%
US $40.001-$60.000	3	5.45%
US $60.001-80.000	9	16.36%
US $80.001-100.000	10	18.18%
US $100.001-120.000	2	3.64%
US $120.001-140.000	1	1.82%
US $140.001-160.000	3	5.45%
More than $160.000	4	7.27%
**Total**	**55**	**100.00%**
**People in household**		
1	6	10.91%
2	7	12.73%
3	12	21.82%
4	21	38.18%
5	7	12.73%
6	2	3.64%
**Total**	**55**	**100.00%**
**Place of residence**		
Large city or urban area	34	61.82%
Rural area NOT on a farm or ranch	15	27.27%
Rural area on a farm or ranch	6	10.91%
**Total**	**55**	**100.00%**
