# Supplementary material for: Explicit Not Implicit Preferences Predict Conservation Intentions for Endangered Species and Biomes
Source: PLoS One. 2017 Jan 30;12(1):e0170973. doi: 10.1371/journal.pone.0170973 (PMC5279788; doi:10.1371/journal.pone.0170973)
Supplement: S2 Table — (PDF) [file pone.0170973.s002.pdf]

S2 Table. Participant demographics for study 2.

Variable	*n*	Percentage
**Ethnicity**		
White or Caucasian	12	21.05%
Japanese	3	5.26%
Chinese	29	50.88%
Filipino	1	1.75%
Middle Eastern	1	1.75%
South Asian (from India, Bangladesh, Pakistan, etc)	2	3.51%
Other	1	1.75%
Multiracial	8	14.04%
**Total**	**57**	**100.00%**
**Highest level of education completed**		
High school or equivalent	51	89.47%
College	2	3.51%
Bachelor’s degree	2	3.51%
Master’s degree	1	1.75%
Doctoral degree	1	1.75%
**Total**	**57**	**100.00%**
**Employment (Select more than one)**		
Student	54	
Unemployed	11	
Agriculture, forestry, fishing, hunting	1	
Arts, entertainment, recreation	5	
Education College/University	1	
Education Primary/Secondary	2	
Finance and insurance	1	
Business, marketing, administration	12	
Health Care, social assistance	5	
Scientific or technical services	1	
Construction	1	
Other	9	
**Religious affiliation**		
Muslim	1	1.75%
Buddhist	2	3.51%
Hindu	1	1.75%
Catholic	10	17.54%
Protestant	3	5.26%
Jewish	1	1.75%
Atheist	11	19.30%

**Variable**	***n***	**Percentage**
Agnostic	11	19.30%
Other	17	29.82%
**Total**	**57**	**100.00%**
**Annual household income**		
Less than US$20.000	10	17.54%
US $20.001-$40.000	4	7.02%
US $40.001-$60.000	13	22.81%
US $60.001-80.000	7	12.28%
US $80.001-100.000	9	15.79%
US $100.001-120.000	2	3.51%
US $120.001-140.000	5	8.77%
US $140.001-160.000	3	5.26%
More than $160.000	4	7.02%
**Total**	**57**	**100.00%**
**People in household**		
1	2	3.51%
2	3	5.26%
3	16	28.07%
4	23	40.35%
5	11	19.30%
6	1	1.75%
7	1	1.75%
**Total**	**57**	**100.00%**
**Place of residence**		
Large city or urban area	32	56.24%
Rural area NOT on a farm or ranch	1	1.75%
Rural area on a farm or ranch	2	3.51%
Small city	3	5.26%
Suburban area	19	33.33%
**Total**	**57**	**100.00%**
