# Supplementary material for: Explicit Not Implicit Preferences Predict Conservation Intentions for Endangered Species and Biomes
Source: PLoS One. 2017 Jan 30;12(1):e0170973. doi: 10.1371/journal.pone.0170973 (PMC5279788; doi:10.1371/journal.pone.0170973)
Supplement: S3 Table — (PDF) [file pone.0170973.s003.pdf]

S3 Table. Participant demographics for study 3.

Variable	*n*	Percentage
**Ethnicity**		
White or Caucasian	287	61.99%
Black or African-American	29	6.26%
Hispanic or Latino (includes Mexican, Central American and South American)	14	3.02%
Korean	5	1.08%
Japanese	1	0.22%
Chinese	2	0.43%
Filipino	1	0.22%
Pacific islander	1	0.22%
Middle eastern	1	0.22%
South Asian (from India, Bangladesh, Pakistan, etc)	98	21.17%
Other	5	1.08%
Multiracial	19	4.10%
**Total**	**463**	**100.00%**
**Highest level of education completed**		
High school or equivalent	80	17.28%
Vocational/Technical school	23	4.97%
College	83	17.93%
Bachelor's degree	185	39.96%
Professional degree (MD, JD, etc)	19	4.10%
Master's degree	62	13.39%
Doctoral degree	8	1.73%
Other	3	0.65%
**Total**	**463**	**100.00%**
**Employment (Select more than one)**		
Student	47	
Unemployed	76	
Agriculture, forestry, fishing, hunting	12	
Arts, entertainment, recreation	31	
Design/publicity	9	
Education College/University	26	
Education Primary/Secondary	24	
Finance and insurance	32	

Variable	*n*	Percentage
Business, marketing, administration	54	
Government and public administration	16	
Health Care, social assistance	34	
Legal services	7	
Scientific or technical services	22	
Software	40	
Transportation	16	
Construction	16	
Manufacturing	16	
Other	72	
**Religious affiliation**		
Mormon	4	0.86%
Muslim	14	3.02%
Orthodox Church such as Greek or Russian Orthodox Church	3	0.65%
Buddhist	6	1.30%
Catholic	75	16.20%
Protestant	86	18.57%
Jewish	7	1.51%
Jehovah's Witness	5	1.08%
Hindu	72	15.55%
Atheist	57	12.31%
Agnostic	83	17.93%
Other	51	11.02%
**Total**	**463**	**100.00%**
**Annual household income**		
Less than US$20.000	127	27.43%
US $20.001-$40.000	122	26.35%
US $40.001-$60.000	86	18.57%
US $60.001-80.000	51	11.02%
US $80.001-100.000	37	7.99%
US $100.001-120.000	16	3.46%
US $120.001-140.000	11	2.38%
US $140.001-160.000	4	0.86%
More than $160.000	9	1.94%
**Total**	**463**	**100.00%**
**People in household**		
1	95	20.52%

**Variable**	***n***	**Percentage**
2	111	23.97%
3	106	22.89%
4	73	15.77%
5	54	11.66%
6	17	3.67%
7	3	0.65%
8	3	0.65%
11	1	0.22%
**Total**	**463**	**100.00%**
**Place of residence**		
Large city or urban area	145	31.32%
Rural area NOT on a farm or ranch	132	28.51%
Rural area on a farm or ranch	126	27.21%
Small city or town	22	4.75%
Suburban area	38	8.21%
**Total**	**463**	**100.00%**
