# Supplementary material for: Explicit Not Implicit Preferences Predict Conservation Intentions for Endangered Species and Biomes
Source: PLoS One. 2017 Jan 30;12(1):e0170973. doi: 10.1371/journal.pone.0170973 (PMC5279788; doi:10.1371/journal.pone.0170973)
Supplement: S4 Table — (PDF) [file pone.0170973.s004.pdf]

S4 Table. Creative commons licenses for the pictures used in the MC-IAT of species and biomes surveys in studies 1, 2 and 3.

The pictures presented in this supplementary file were the pictures used in the IAT instruction page. Pictures were numbered from left to right as they appear in the instruction page for each species.

Picture	Author	License
Caribou_1	Josh More	CC BY-NC-ND 2.0
Caribou_2	Amanda Graham	CC BY-NC-ND 2.0
Caribou_3	Ianqui Doodle	CC BY-NC-ND 2.0
Caribou_4	Travis	CC BY-NC 2.0
Sea_otter_1	Chuq von Rospach	With permission
Sea_otter_2	Chuq von Rospach	With permission
Sea_otter_3	Chuq von Rospach	With permission
Sea_otter_4	Chuq von Rospach	With permission
American_badger_1	Jon Nelson	CC BY 2.0
American_badger_2	Yathin	CC BY-NC-ND 2.0
American_badger_3	Jerry Oldenetell	CC BY-NC-SA 2.0
American_badger_4	Bethany Weeks	CC BY-NC-SA 2.0
Yellow_breasted_chat_1	HarmonyonPlanetEarth	CC BY 2.0
Yellow_breasted_chat_2	HarmonyonPlanetEarth	CC BY 2.0
Yellow_breasted_chat_3	Kelly Colgan Azar	CC BY-ND 2.0
Yellow_breasted_chat_4	Budgora	CC BY-NC-ND 2.0
Tundra_1	David Mark	CC BY 1.0
Tundra_2	Billy Lindblom	CC BY 2.0
Tundra_3	Bryce Bradford	CC BY-NC-ND 2.0
Tundra_4	Mark Dries	CC BY-NC-ND 2.0
Forest_1	Maurizio Agelli	CC BY-NC-ND 2.0
Forest_2	H.-D. Viktor Boehm	CC BY 2.0
Forest_3	Mmartinsson	CC BY-NC-ND 2.0
Forest_4	Roman Boed	CC BY 2.0
Ocean_1	Carrie Johnson	CC BY-ND 2.0
Ocean_2	Craig	CC BY 2.0
Ocean_3	Dan Oestreich	CC BY-NC-ND 2.0
Ocean_4	Joyce Pedersen	CC BY-NC 2.0
Grassland_1	Andy	CC BY-NC-ND 2.0
Grassland_2	Alexander Stinhof	CC BY-NC-ND 2.0
Grassland_3	Alan Schmierer	CC0 1.0
Grassland_4	Wallpaper	CC BY 2.0
