# Supplementary material for: Explicit Not Implicit Preferences Predict Conservation Intentions for Endangered Species and Biomes
Source: PLoS One. 2017 Jan 30;12(1):e0170973. doi: 10.1371/journal.pone.0170973 (PMC5279788; doi:10.1371/journal.pone.0170973)
Supplement: S1 Fig — (PDF) [file pone.0170973.s005.pdf]

S1 Fig. Instruction page for the MC-IAT for Study 1 and 2.

In this study, you will see 4 categories of animals. Please memorize these images of animals:

Caribou

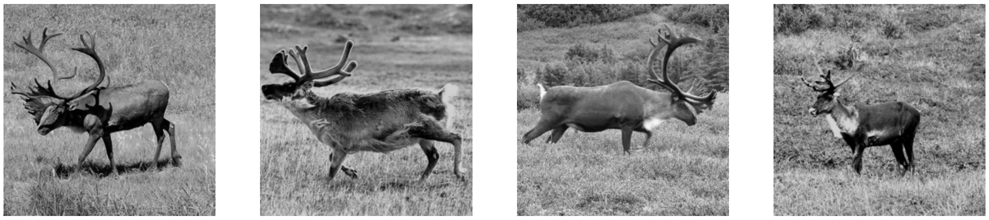

Sea otter

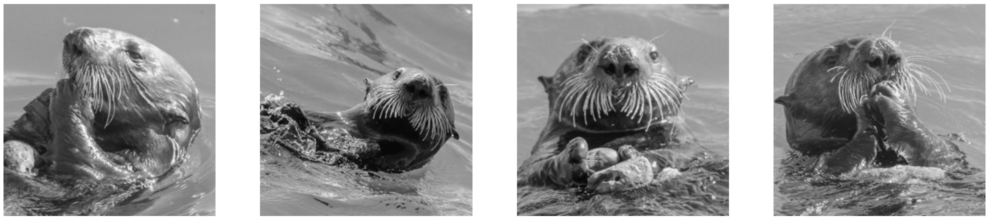

American badger

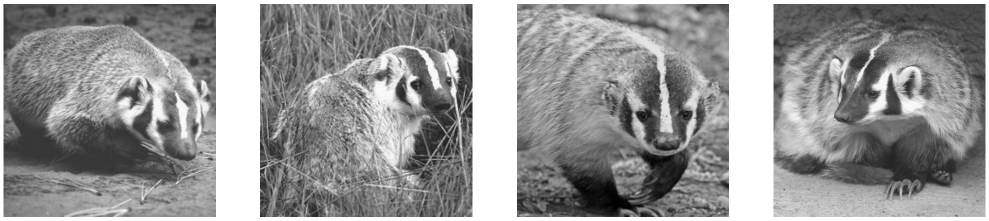

Yellow-breasted chat

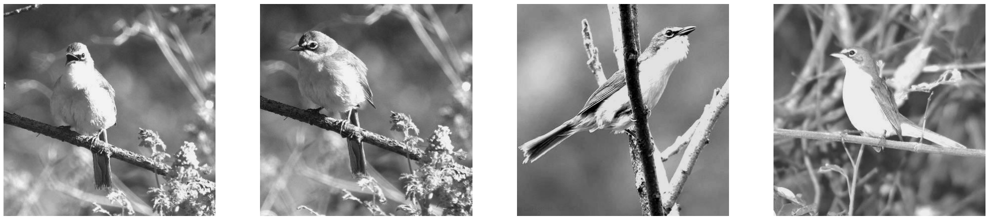

You will also see 4 good words and 4 bad words. Please memorize these words:

Good words: LOVE, PLEASANT, GREAT, WONDERFUL
Bad words: HATE, UNPLEASANT, AWFUL, TERRIBLE

You will press the right "I" key if you see **Good words** and images from one category. You will press the left "E" key for other images and words. If you respond incorrectly, you will a "WRONG" sign. We will start with some practice to help you get used to the task. Please classify words and images as quickly as you can while making as few mistakes as possible.

In this study, you will see 4 categories of environments. Please memorize these images of environments:

Forest

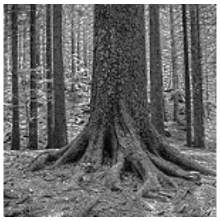 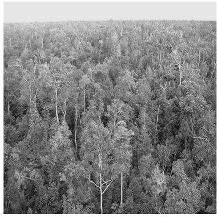 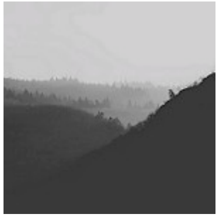 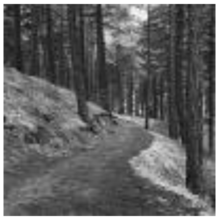

Grassland

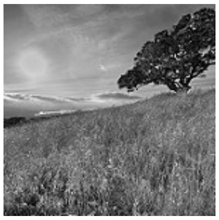 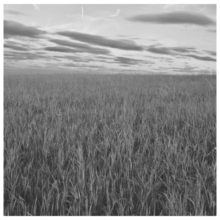 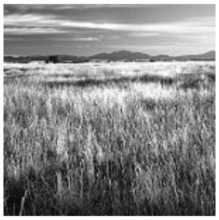 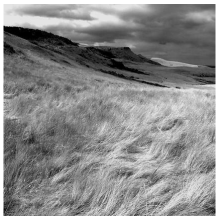

Ocean

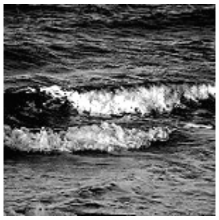 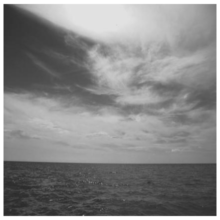 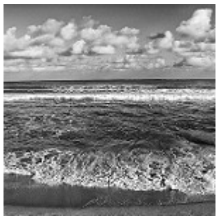 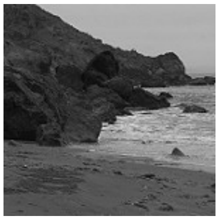

Tundra

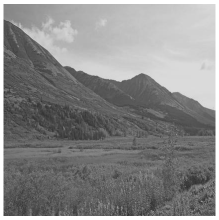 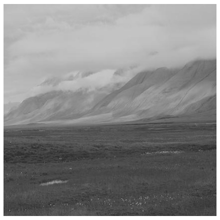 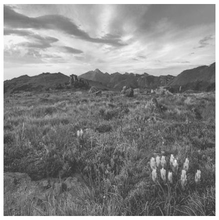 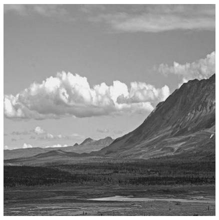

You will also see 4 good words and 4 bad words. Please memorize these words:

Good words: LOVE, PLEASANT, GREAT, WONDERFUL
Bad words: HATE, UNPLEASANT, AWFUL, TERRIBLE

You will press the right “I” key if you see **Good words** and images from one category. You will press the left "E" key for other images and words. If you respond incorrectly, you will a "WRONG" sign. We will start with some practice to help you get used to the task. Please classify words and images as quickly as you can while making as few mistakes as possible.
