# Supplementary material for: Explicit Not Implicit Preferences Predict Conservation Intentions for Endangered Species and Biomes
Source: PLoS One. 2017 Jan 30;12(1):e0170973. doi: 10.1371/journal.pone.0170973 (PMC5279788; doi:10.1371/journal.pone.0170973)
Supplement: S2 Fig — (PDF) [file pone.0170973.s006.pdf]

S2 Fig. Questionnaire for study 1.

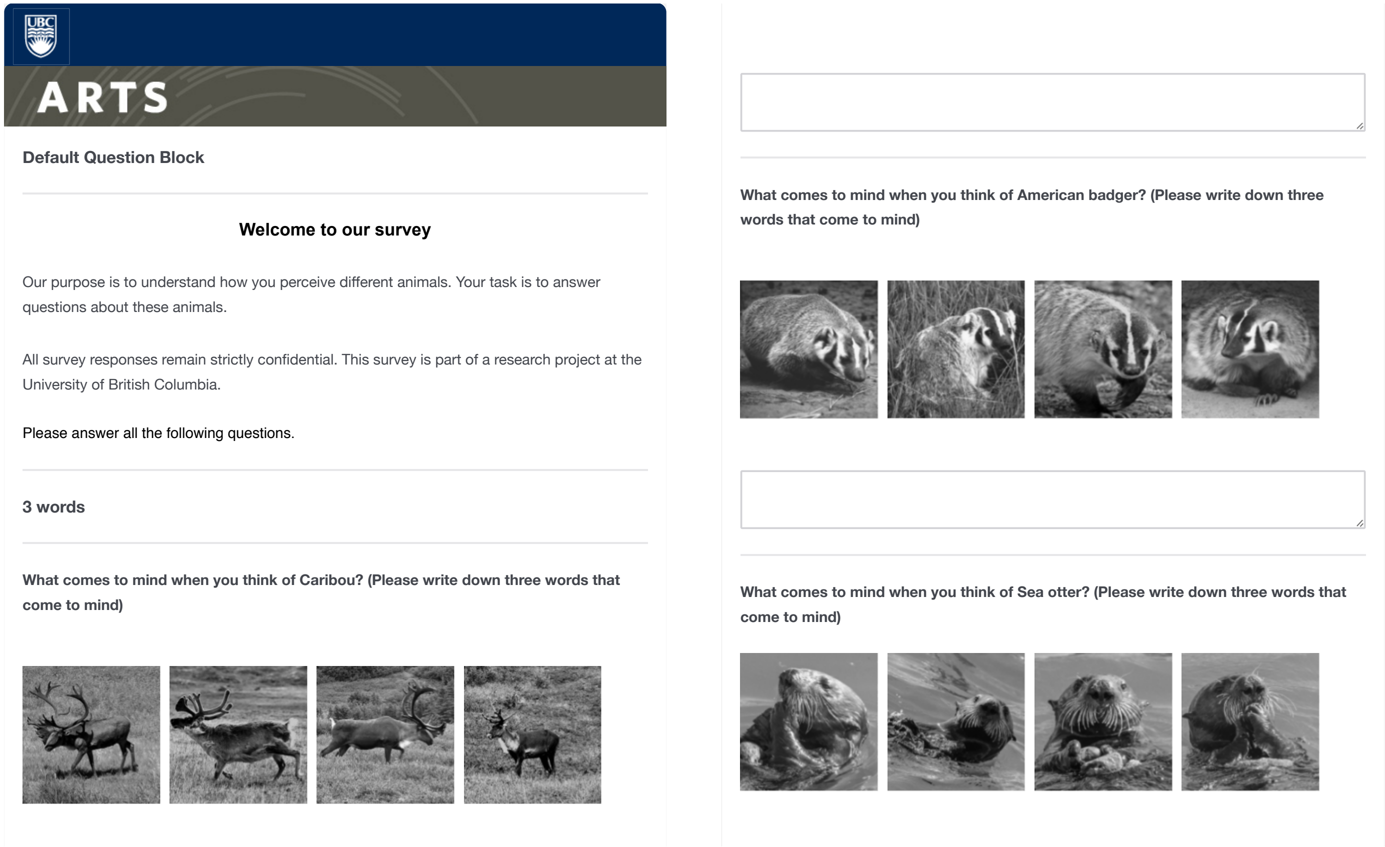

ARTS

Default Question Block

Welcome to our survey

Our purpose is to understand how you perceive different animals. Your task is to answer questions about these animals.

All survey responses remain strictly confidential. This survey is part of a research project at the University of British Columbia.

Please answer all the following questions.

3 words

What comes to mind when you think of Caribou? (Please write down three words that come to mind)

What comes to mind when you think of American badger? (Please write down three words that come to mind)

What comes to mind when you think of Sea otter? (Please write down three words that come to mind)

What comes to mind when you think of Yellow-breasted chat? (Please write down three words that come to mind)

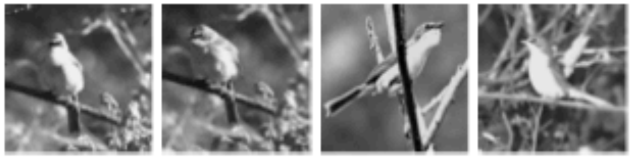

Donation

How much money (in Canadian dollars) are you willing to donate to conserve each of these animals?

0 American badger

0 Caribou

0 Sea otter

0 Yellow-breasted chat

Like/Familiarity

Please answer the following questions

	Not at all										Extremely
	0	1	2	3	4	5	6	7	8	9	10
How much do you like **Caribou?**											
How familiar are you with **Caribou?**											

Please answer the following questions

	Not at all										Extremely
	0	1	2	3	4	5	6	7	8	9	10
How much do you like **American badger?**											
How familiar are you with **American badger?**											

Please answer the following questions

	Not at all										Extremely
	0	1	2	3	4	5	6	7	8	9	10
How much do you like **Sea otter?**											

How familiar are you with **Sea otter?**

Please answer the following questions

	Not at all										Extremely
	0	1	2	3	4	5	6	7	8	9	10
How much do you like **Yellow-breasted chat?**											
How familiar are you with **Yellow-breasted chat?**											

Ranking favorites

Rank these animals (by dragging and dropping) in order of your most (1) to least (4) favorite

American badger

Caribou

Sea otter

Yellow-breasted chat

Ranking endangered

Rank these animals (by dragging and dropping) in order of the most (1) to least (4) endangered

American badger

Caribou

Sea otter

Yellow-breasted chat

Conservation behaviour

Have you ever been a member of a conservation, animal welfare, or other environmental organization?

Yes

No

Do you have pets?

Yes

No

Have you had pets in the past?

Yes

No

Have you ever gone hunting?

Yes
No

Have you ever gone fishing?

Yes
No

How much time do you spend in nature (public parks, national or provincial parks, forests, etc) on a regular **weekday**?

None
Less than 1/2 an hour
About 1/2 an hour
About 1 hour
2-3 hours
4 or more hours

How much time do you spend in nature (public parks, national or provincial parks, forests, etc) on a regular **weekend day**?

None
Less than 1/2 an hour
About 1/2 an hour
About 1 hour
2-3 hours
4 or more hours

Instructions for picture ranking

The following questions will have images of various animals for you to rate. Please provide your judgements as truthfully as possible.

Here is one example of the image you will be rating. There are 5 questions below this image and please provide your judgements using the scales (0 = "not at all", 10 = "extremely").

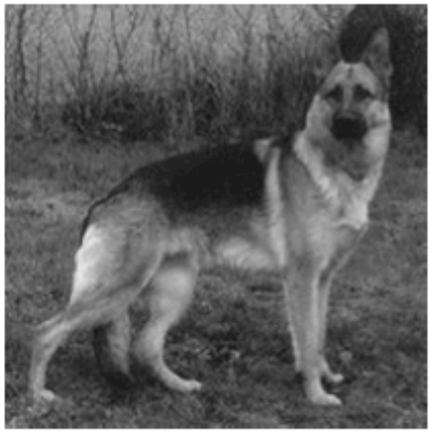

	Not at all										Extremely
	0	1	2	3	4	5	6	7	8	9	10
How beautiful do you think this picture is?											
How much do you like this image?											
How much do you like this animal?											

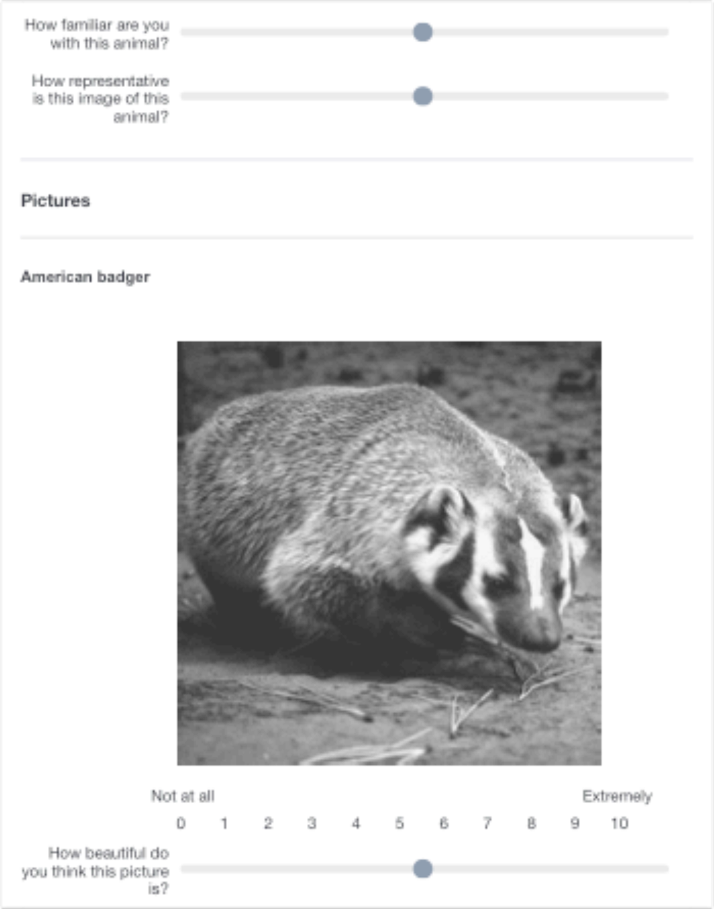
How familiar are you with this animal?
How representative is this image of this animal?
Pictures
American badger
Not at all
Extremely
0 1 2 3 4 5 6 7 8 9 10
How beautiful do you think this picture is?

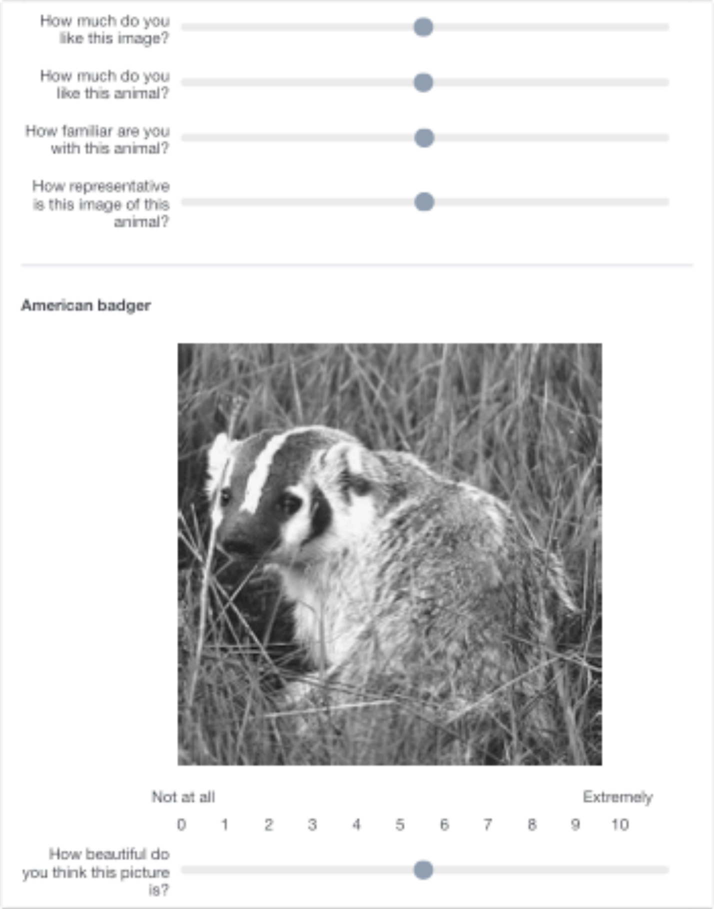
How much do you like this image?
How much do you like this animal?
How familiar are you with this animal?
How representative is this image of this animal?
American badger
Not at all
Extremely
0 1 2 3 4 5 6 7 8 9 10
How beautiful do you think this picture is?

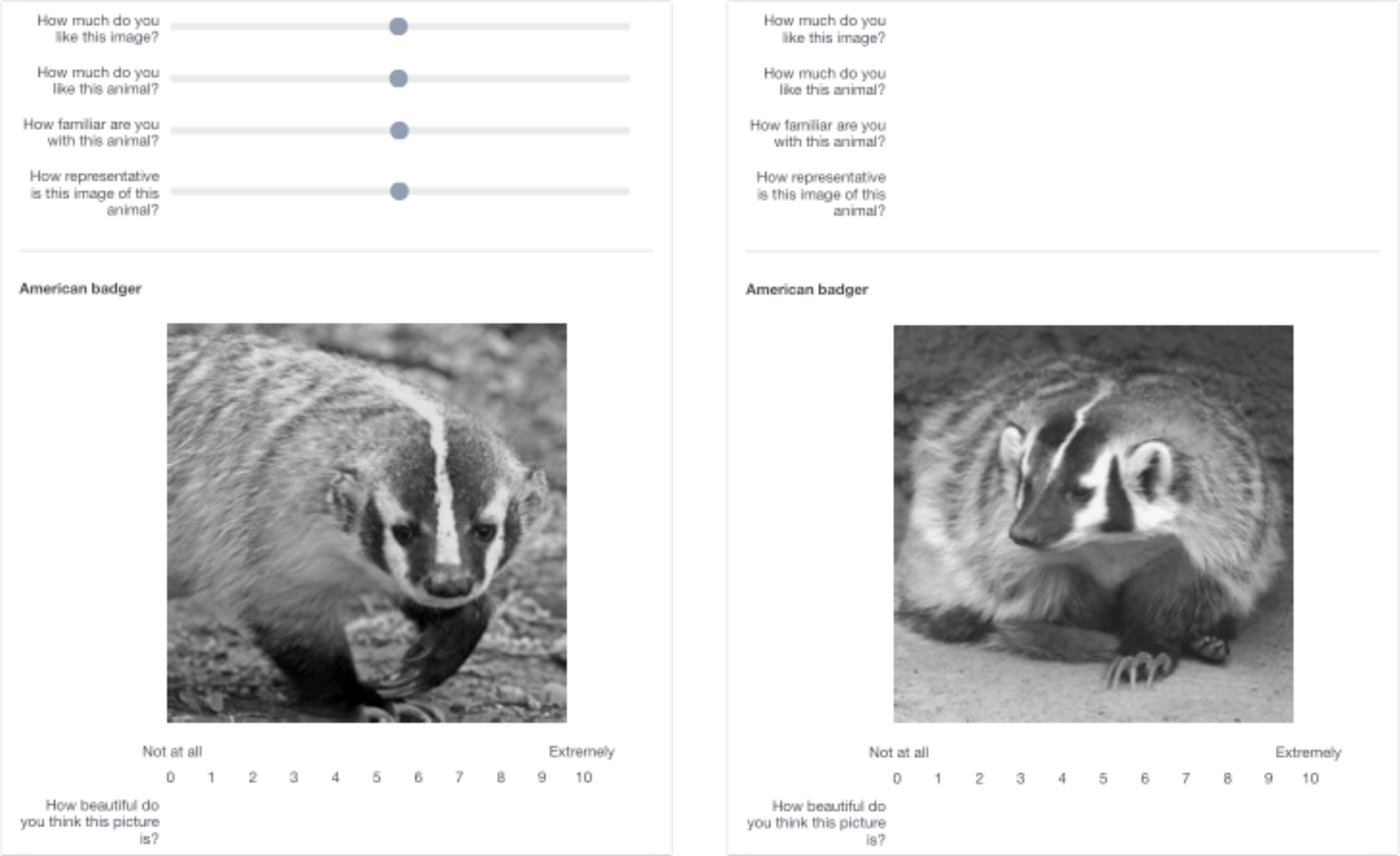
How much do you like this image?
How much do you like this animal?
How familiar are you with this animal?
How representative is this image of this animal?
American badger
Not at all
Extremely
0 1 2 3 4 5 6 7 8 9 10
How beautiful do you think this picture is?
How much do you like this image?
How much do you like this animal?
How familiar are you with this animal?
How representative is this image of this animal?
American badger
Not at all
Extremely
0 1 2 3 4 5 6 7 8 9 10
How beautiful do you think this picture is?

How much do you like this image?

How much do you like this animal?

How familiar are you with this animal?

How representative is this image of this animal?

Caribou

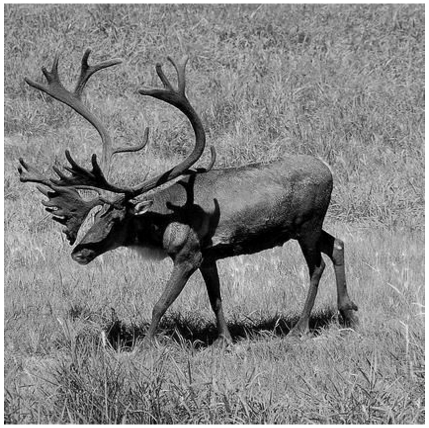

Not at all										Extremely
0	1	2	3	4	5	6	7	8	9	10

How beautiful do you think this picture is?

How much do you like this image?

How much do you like this animal?

How familiar are you with this animal?

How representative is this image of this animal?

Caribou

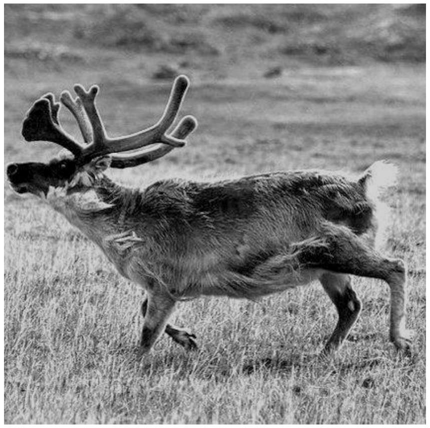

Not at all										Extremely
0	1	2	3	4	5	6	7	8	9	10

How beautiful do you think this picture is?

How much do you like this image?

How much do you like this animal?

How familiar are you with this animal?

How representative is this image of this animal?

Caribou

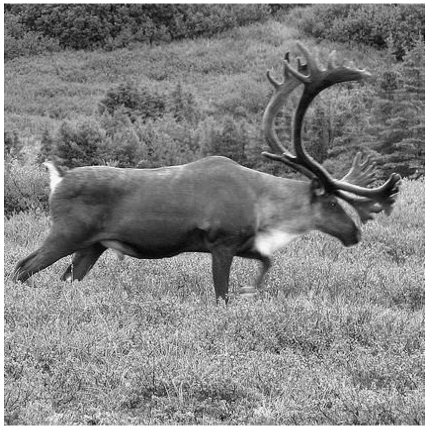

Not at all										Extremely
0	1	2	3	4	5	6	7	8	9	10

How beautiful do you think this picture is?

How much do you like this image?

How much do you like this animal?

How familiar are you with this animal?

How representative is this image of this animal?

Caribou

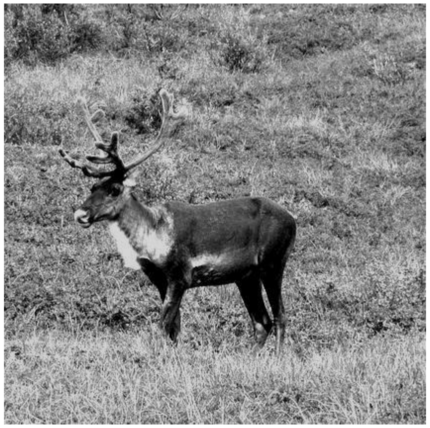

Not at all										Extremely
0	1	2	3	4	5	6	7	8	9	10

How beautiful do you think this picture is?

How much do you like this image?

How much do you like this animal?

How familiar are you with this animal?

How representative is this image of this animal?

Sea otter

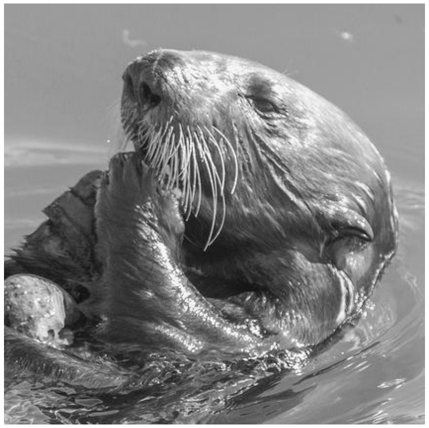

Not at all Extremely

0 1 2 3 4 5 6 7 8 9 10

How beautiful do you think this picture is?

How much do you like this image?

How much do you like this animal?

How familiar are you with this animal?

How representative is this image of this animal?

Sea otter

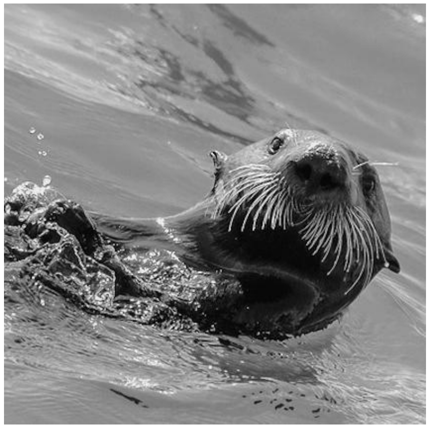

Not at all Extremely

0 1 2 3 4 5 6 7 8 9 10

How beautiful do you think this picture is?

How much do you like this image?

How much do you like this animal?

How familiar are you with this animal?

How representative is this image of this animal?

Sea otter

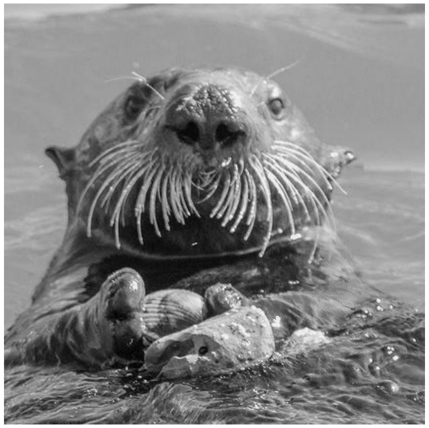

Not at all Extremely

0 1 2 3 4 5 6 7 8 9 10

How beautiful do you think this picture is?

How much do you like this image?

How much do you like this animal?

How familiar are you with this animal?

How representative is this image of this animal?

Sea otter

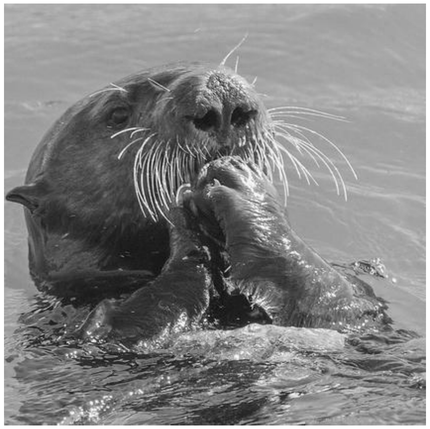

Not at all Extremely

0 1 2 3 4 5 6 7 8 9 10

How beautiful do you think this picture is?

How much do you like this image?

How much do you like this animal?

How familiar are you with this animal?

How representative is this image of this animal?

Yellow-breasted chat

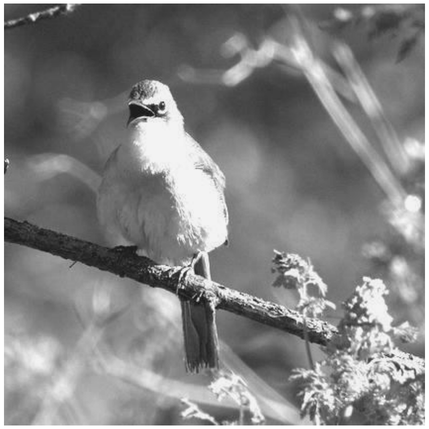

Not at all | Extremely

0 1 2 3 4 5 6 7 8 9 10

How beautiful do you think this picture is?

How much do you like this image?

How much do you like this animal?

How familiar are you with this animal?

How representative is this image of this animal?

Yellow-breasted chat

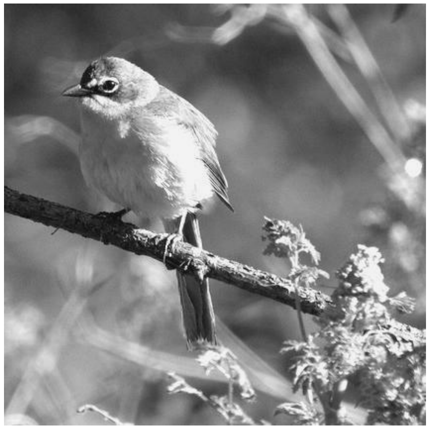

Not at all | Extremely

0 1 2 3 4 5 6 7 8 9 10

How beautiful do you think this picture is?

How much do you like this image?

How much do you like this animal?

How familiar are you with this animal?

How representative is this image of this animal?

Yellow-breasted chat

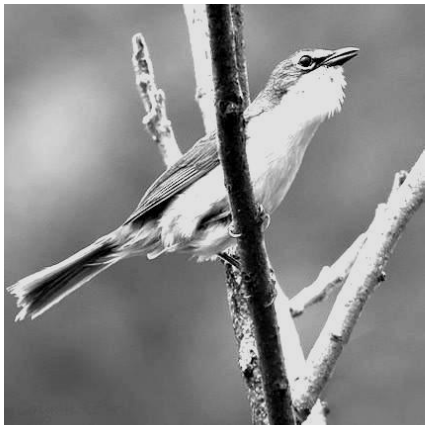

Not at all										Extremely
0	1	2	3	4	5	6	7	8	9	10

How beautiful do you think this picture is?

How much do you like this image?

How much do you like this animal?

How familiar are you with this animal?

How representative is this image of this animal?

Yellow-breasted chat

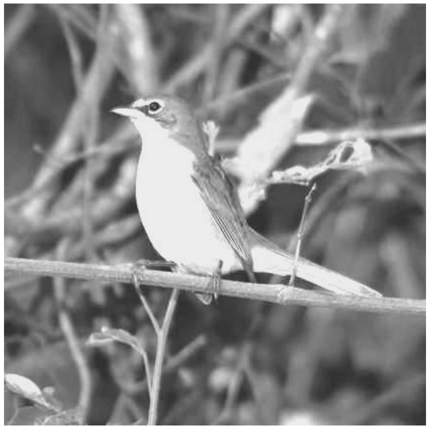

Not at all										Extremely
0	1	2	3	4	5	6	7	8	9	10

How beautiful do you think this picture is?

How much do you like this image?

How much do you like this animal?

How familiar are you with this animal?

How representative is this image of this animal?

Demographics

In this last section of the survey, we would like to learn more about your background and your current household characteristics. You can be assured that all your answers will be kept confidential. This information will only be used to report results among groups of people. We will never identify individuals or households with these responses.

Please write down your full name as it appears on the HSP.

Please write down your email address as it appears on the HSP.

Age (in years)

Gender

Male
Female
Other

Where do you live? (City or town, Country)

Where are you from? (City or town, Country)

What races or ethnic background do you consider yourself to be? Please check all that apply

White or Caucasian
Black or African-American
Hispanic or Latino (includes Mexican, Central American and South American)
Korean
Japanese
Chinese
Filipino
Pacific islander
Middle eastern
African (NOT African-American)
South Asian (from India, Bangladesh, Pakistan, etc)
Other

What is your religious affiliation?

Mormon
Muslim
an Orthodox Church such as Greek or Russian Orthodox Church
Buddhist
Catholic
Protestant
Jewish
Jehovah's Witness
Hindu
Atheist
Agnostic
Other

What is your political orientation? Rate from a scale from -5= very liberal (left wing) to 5= very conservative (right wing)

	Left wing										Right wing
	-5	-4	-3	-2	-1	0	1	2	3	4	5
Political orientation											

What is the highest level of education you have completed?

High school or equivalent
Vocational/Technical school
College
Bachelor's degree
Professional degree (MD, JD, etc)
Master's degree
Doctoral degree
Other

Which of the following categories best describes your area of employment (regardless of your actual position)? Check all that apply

Student
Unemployed
Agriculture, forestry, fishing, hunting
Arts, entertainment, recreation
Design/publicity
Education College/University
Education Primary/Secondary
Finance and insurance
Business, marketing, administration
Government and public administration
Health Care, social assistance
Legal services
Scientific or technical services
Software
Transportation
Construction
Manufacturing
Other

Please indicate your total annual household income (in Canadian Dollars)

Less than $20.000
$20.001-$40.000
$40.001-$60.000
$60.001-80.000
$80.001-100.000
$100.001-120.000

$120.001-140.000
$140.001-160.000
More than $160.000

How many people live in your household including you?

What do you consider your place of residence to be?

Large city or urban area
Suburban area
Small city or town
Rural area on a farm or ranch
Rural area NOT on a farm or ranch

Thank you for participating in this survey!

Powered by Qualtrics
