# Supplementary material for: Explicit Not Implicit Preferences Predict Conservation Intentions for Endangered Species and Biomes
Source: PLoS One. 2017 Jan 30;12(1):e0170973. doi: 10.1371/journal.pone.0170973 (PMC5279788; doi:10.1371/journal.pone.0170973)
Supplement: S3 Fig — (PDF) [file pone.0170973.s007.pdf]

S3 Fig. Questionnaire for study 2.

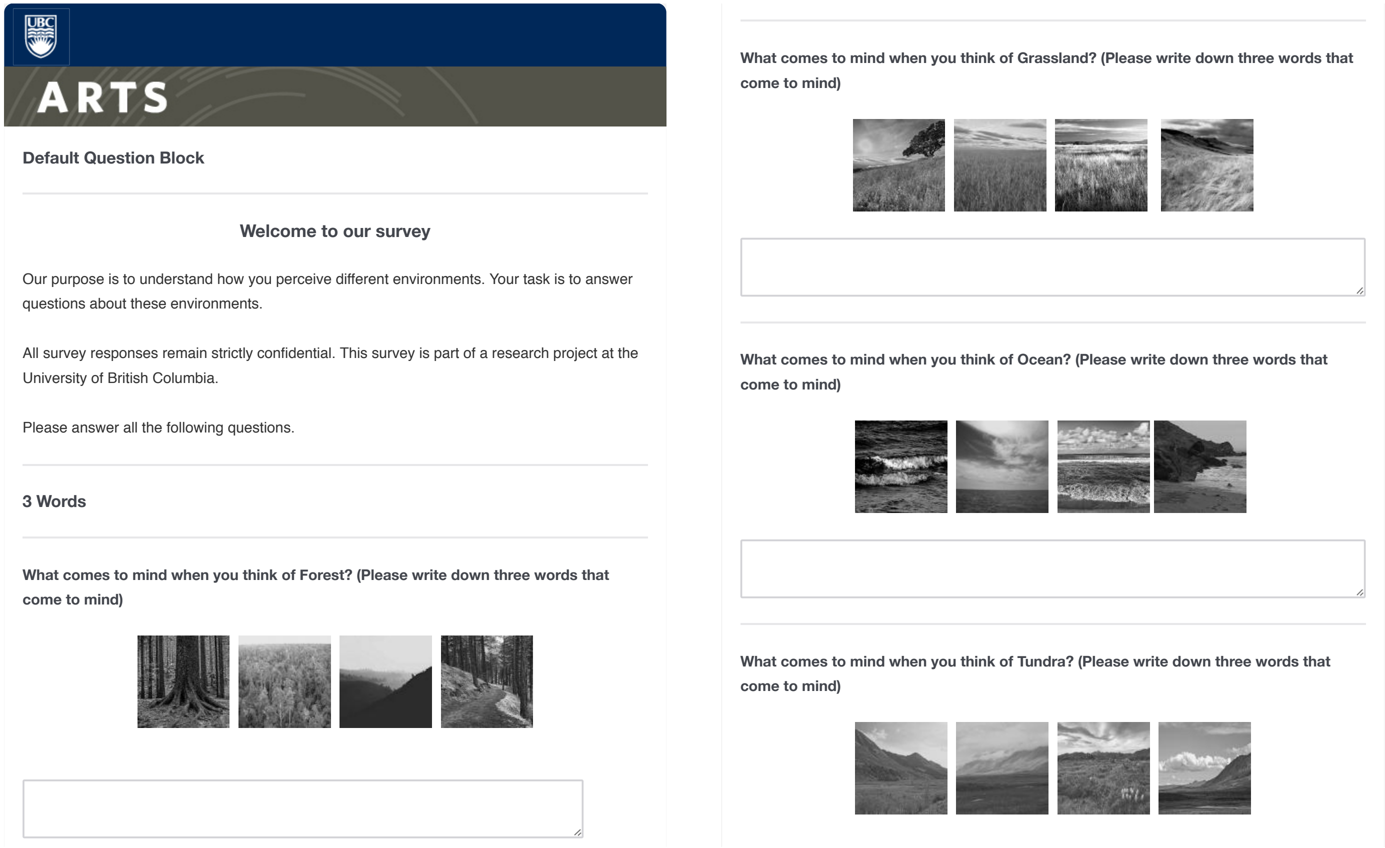

UBC

ARTS

Default Question Block

Welcome to our survey

Our purpose is to understand how you perceive different environments. Your task is to answer questions about these environments.

All survey responses remain strictly confidential. This survey is part of a research project at the University of British Columbia.

Please answer all the following questions.

3 Words

What comes to mind when you think of Forest? (Please write down three words that come to mind)

What comes to mind when you think of Grassland? (Please write down three words that come to mind)

What comes to mind when you think of Ocean? (Please write down three words that come to mind)

What comes to mind when you think of Tundra? (Please write down three words that come to mind)

Donation

How much money (in Canadian dollars) would you give to conserve each of these environments?

- 0 Forest
- 0 Grassland
- 0 Ocean
- 0 Tundra

Like/Familiarity

Please answer the following questions

	Not at all										Extremely
	0	1	2	3	4	5	6	7	8	9	10
How much do you like **forest?**											
How familiar are you with **forest?**											

Please answer the following questions

	Not at all										Extremely
	0	1	2	3	4	5	6	7	8	9	10
How much do you like **grassland?**											
How familiar are you with **grassland?**											

Please answer the following questions

	Not at all										Extremely
	0	1	2	3	4	5	6	7	8	9	10
How much do you like **ocean?**											
How familiar are you with **ocean?**											

Please answer the following questions

	Not at all										Extremely
	0	1	2	3	4	5	6	7	8	9	10
How much do you like **tundra?**											
How familiar are you with **tundra?**											

Ranking favorites

Rank these environments (by dragging and dropping) in order of your most (1) to least (4) favorite

Forest

Grassland

Ocean

Tundra

Ranking Endangered

Rank these environments (by dragging and dropping) in order from the most (1) to least (4) threatened

Forest

Grassland

Ocean

Tundra

Money for Conservation

Have you ever been a member of a conservation, animal welfare, or other environmental organization?

Yes
No

Do you have pets?

Yes
No

Have you had pets in the past?

Yes
No

Have you ever gone hunting?

Yes
No

Have you ever gone fishing?

Yes
No

How much time do you spend in nature (public parks, national or provincial parks, forests, etc) on a regular **weekday**?

None
Less than 1/2 an hour
About 1/2 an hour
About 1 hour
2-3 hours
4 or more hours

How much time do you spend in nature (public parks, national or provincial parks, forests, etc)

on a regular **weekend day**?

None

Less than 1/2 an hour

About 1/2 an hour

About 1 hour

2-3 hours

4 or more hours

Instructions for picture ranking

The following questions will have images of various environments for you to rate. Please provide your judgements as truthfully as possible.

Here is one example of an image you will be rating. There are 5 questions below this image and please provide your judgments using the scales (0 = "not at all", 10 = "extremely").

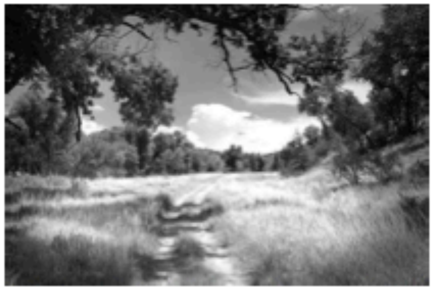

Not at all Extremely

0 1 2 3 4 5 6 7 8 9 10

How beautiful do you think this picture is?

How much do you like this image?

How much do you like this environment?

How familiar are you with this environment?

How representative is this image of this environment?

Pictures

Forest

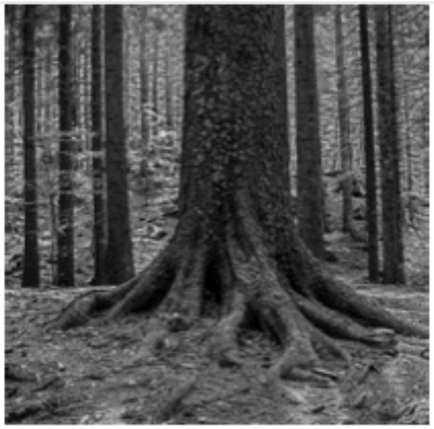

Not at all										Extremely
0	1	2	3	4	5	6	7	8	9	10

How beautiful do you think this picture is?

How much do you like this image?

How much do you like this environment?

How familiar are you with this environment?

How representative of forest is this image?

Forest

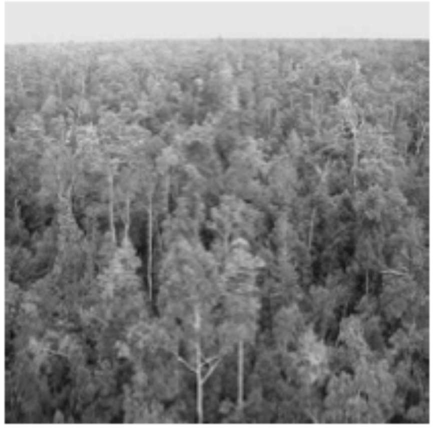

Not at all										Extremely
0	1	2	3	4	5	6	7	8	9	10

How beautiful do you think this picture is?

How much do you like this image?

How much do you like this environment?

How familiar are you with this environment?

How representative of forest is this image?

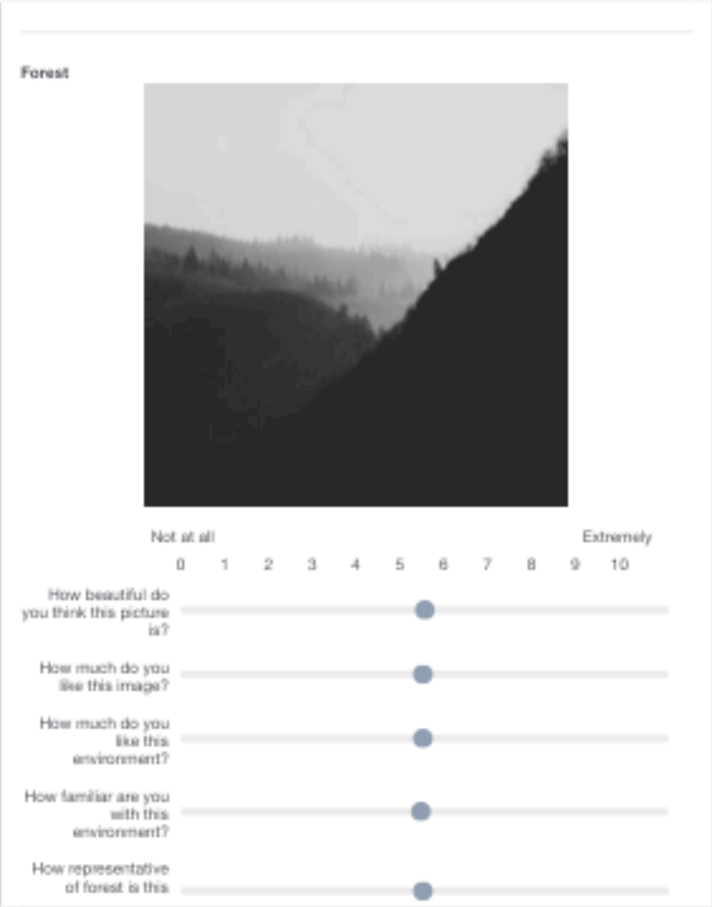
Forest
Not at all
Extremely
0 1 2 3 4 5 6 7 8 9 10
How beautiful do you think this picture is?
How much do you like this image?
How much do you like this environment?
How familiar are you with this environment?
How representative of forest is this

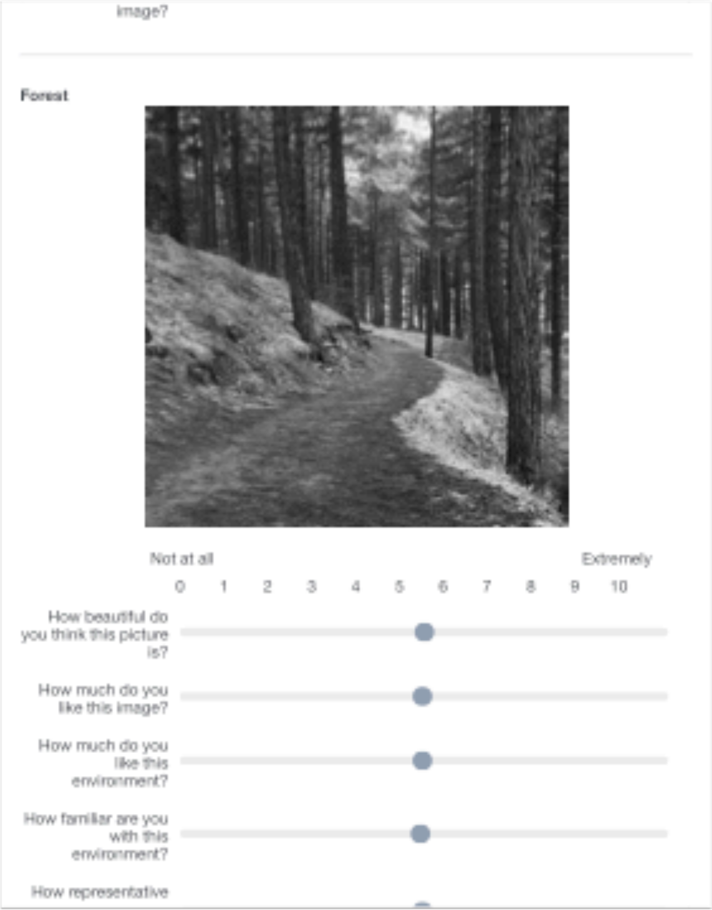
image?
Forest
Not at all
Extremely
0 1 2 3 4 5 6 7 8 9 10
How beautiful do you think this picture is?
How much do you like this image?
How much do you like this environment?
How familiar are you with this environment?
How representative

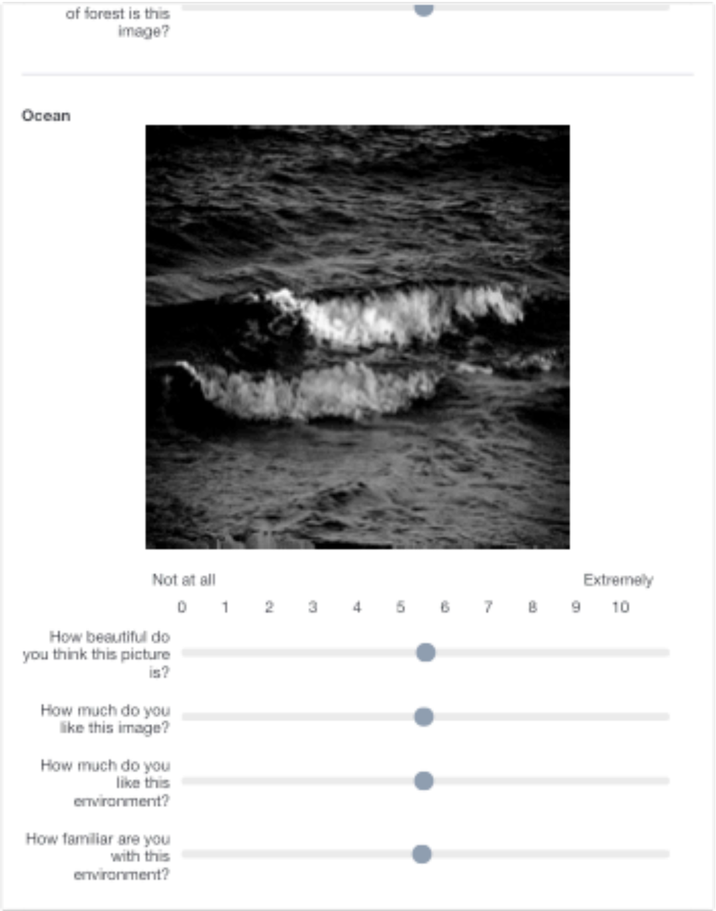
of forest is this image?
Ocean
Not at all
Extremely
0 1 2 3 4 5 6 7 8 9 10
How beautiful do you think this picture is?
How much do you like this image?
How much do you like this environment?
How familiar are you with this environment?

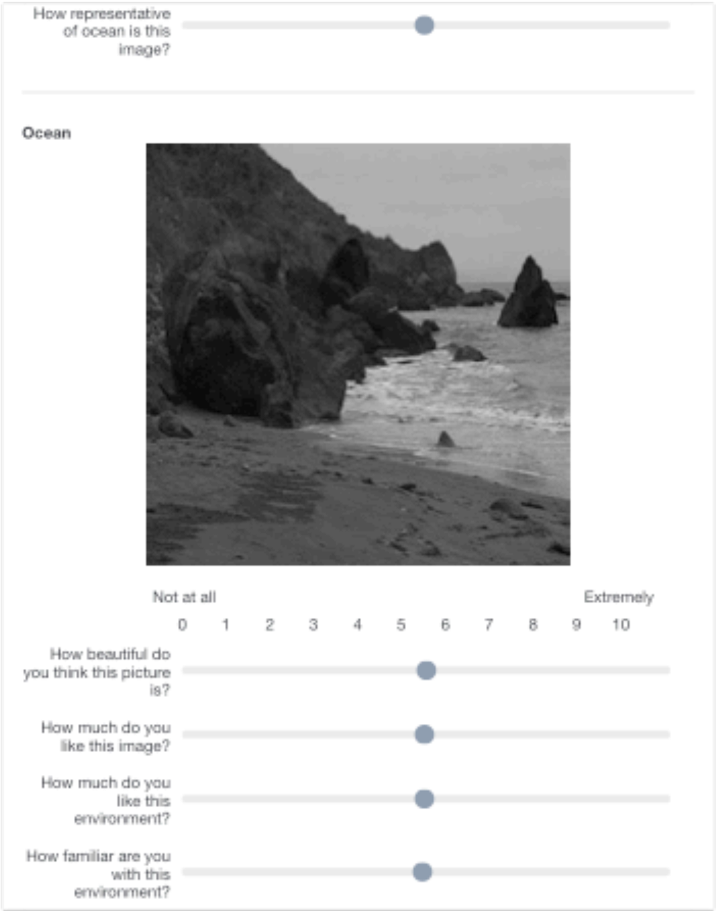
How representative of ocean is this image?
Ocean
Not at all
Extremely
0 1 2 3 4 5 6 7 8 9 10
How beautiful do you think this picture is?
How much do you like this image?
How much do you like this environment?
How familiar are you with this environment?

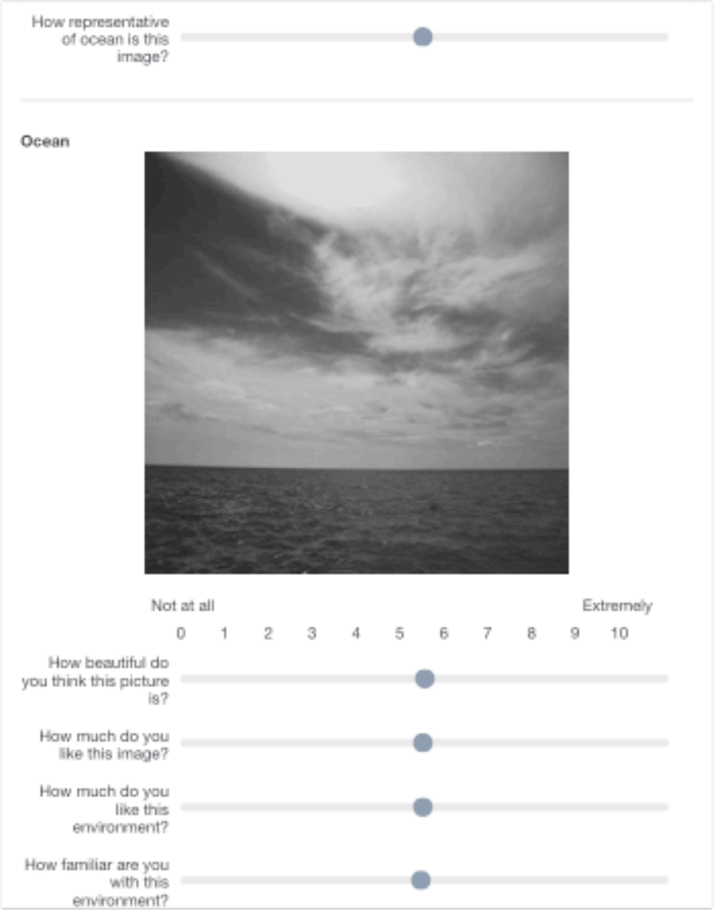
How representative of ocean is this image?
Ocean
Not at all
Extremely
0 1 2 3 4 5 6 7 8 9 10
How beautiful do you think this picture is?
How much do you like this image?
How much do you like this environment?
How familiar are you with this environment?

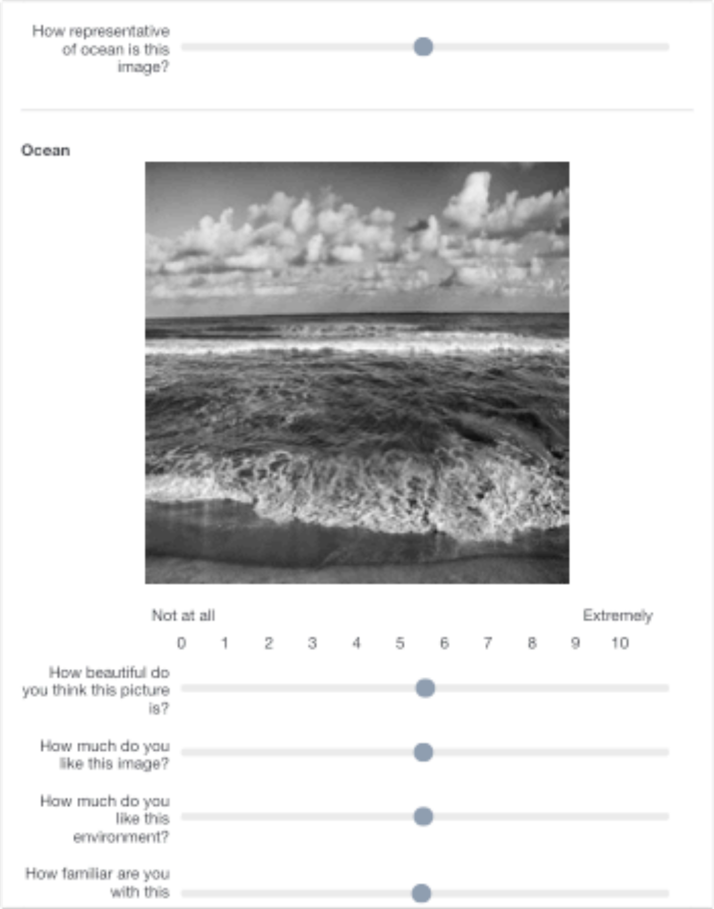
How representative of ocean is this image?
Ocean
Not at all
Extremely
0 1 2 3 4 5 6 7 8 9 10
How beautiful do you think this picture is?
How much do you like this image?
How much do you like this environment?
How familiar are you with this

environment?

How representative of ocean is this image?

Grassland

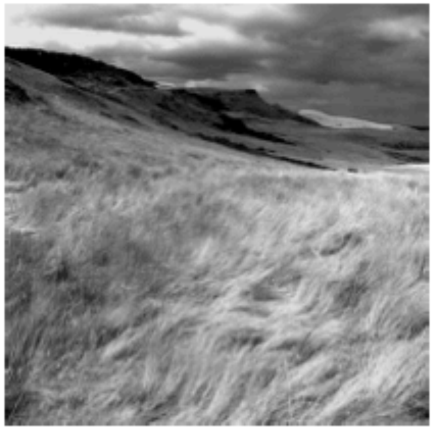

	Not at all									Extremely
	0	1	2	3	4	5	6	7	8	9	10

How beautiful do you think this picture is?

How much do you like this image?

How much do you like this environment?

How familiar are you

with this environment?

How representative of grassland is this image?

Grassland

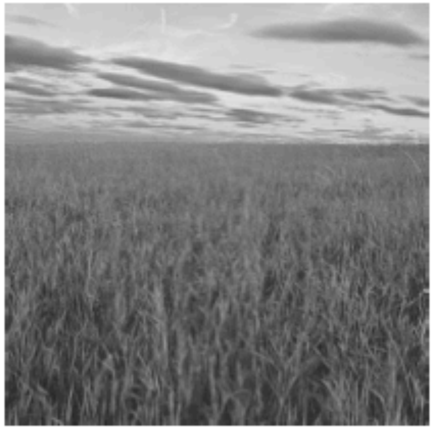

	Not at all									Extremely
	0	1	2	3	4	5	6	7	8	9	10

How beautiful do you think this picture is?

How much do you like this image?

How much do you like this

environment?

How familiar are you with this environment?

How representative of grassland is this image?

Grassland

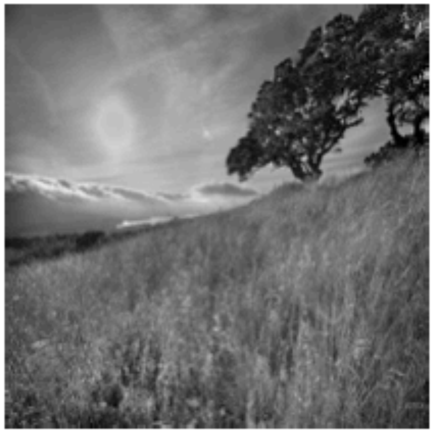

Not at all Extremely

0 1 2 3 4 5 6 7 8 9 10

How beautiful do you think this picture is?

How much do you like this image?

How much do you

like this environment?

How familiar are you with this environment?

How representative of grassland is this image?

Grassland

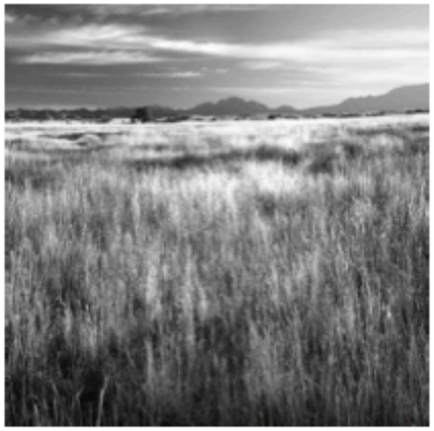

Not at all Extremely

0 1 2 3 4 5 6 7 8 9 10

How beautiful do you think this picture is?

How much do you

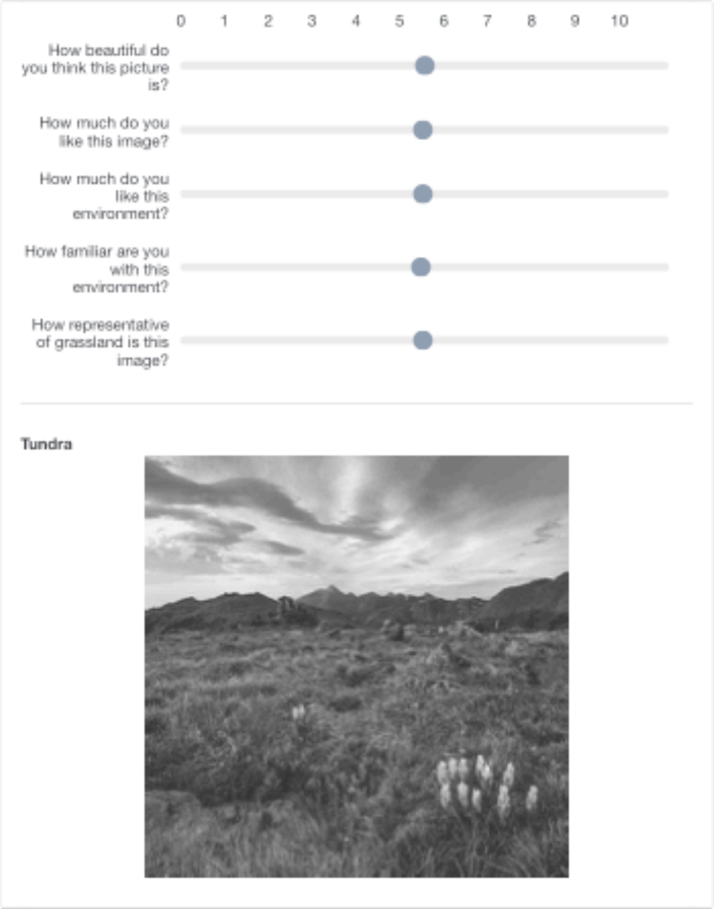
0 1 2 3 4 5 6 7 8 9 10
How beautiful do you think this picture is?
How much do you like this image?
How much do you like this environment?
How familiar are you with this environment?
How representative of grassland is this image?
Tundra

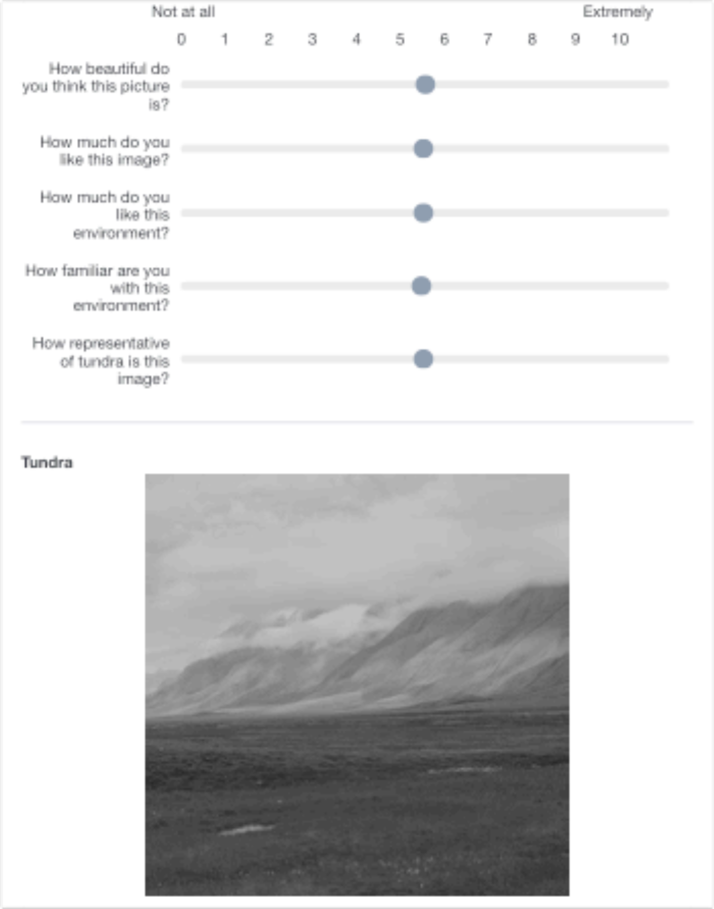
Not at all
Extremely
0 1 2 3 4 5 6 7 8 9 10
How beautiful do you think this picture is?
How much do you like this image?
How much do you like this environment?
How familiar are you with this environment?
How representative of tundra is this image?
Tundra

is?

How much do you like this image?

How much do you like this environment?

How familiar are you with this environment?

How representative of tundra is this image?

Tundra

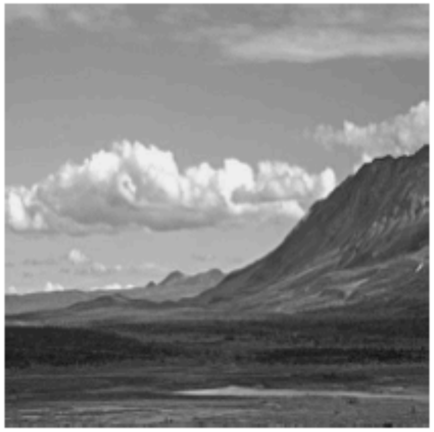

Not at all | Extremely

0 1 2 3 4 5 6 7 8 9 10

How beautiful do

you think this picture is?

How much do you like this image?

How much do you like this environment?

How familiar are you with this environment?

How representative of tundra is this image?

Tundra

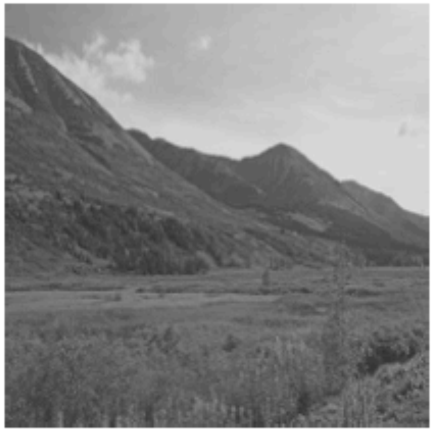

Not at all | Extremely

0 1 2 3 4 5 6 7 8 9 10

How beautiful do you think this picture is?

How much do you like this image?

How much do you like this environment?

How familiar are you with this environment?

How representative of tundra is this image?

Demographics

In this last section of the survey, we would like to learn more about your background and your current household characteristics. You can be assured that all your answers will be kept confidential. This information will only be used to report results among groups of people. We will never identify individuals or households with these responses.

Please write down your full name as it appears on the HSP.

Please write down your email address as it appears on the HSP.

Age (in years)

Gender

Male
Female
Other

Where do you live? (City or town, Country)

Where are you from? (City or town, Country)

What races or ethnic background do you consider yourself to be? Please check all that apply

White or Caucasian
Black or African-American
Hispanic or Latino (includes Mexican, Central American and South American)
Korean
Japanese
Chinese
Filipino
Pacific Islander
Middle Eastern
African (NOT African-American)

South Asian (from India, Bangladesh, Pakistan, etc)
Other

What is your religious affiliation?

Mormon
Muslim
an Orthodox Church such as Greek or Russian Orthodox Church
Buddhist
Catholic
Protestant
Jewish
Jehovah's Witness
Hindu
Atheist
Agnostic
Other

What is your political orientation? Rate from a scale from -5= very liberal (left wing) to 5= very conservative (right wing)

Left Wing Right Wing

-5 -4 -3 -2 -1 0 1 2 3 4 5

Political Orientation

What is the highest level of education you have completed?

High school or equivalent
Vocational/Technical school
College
Bachelor's degree
Professional degree (MD, JD, etc)
Master's degree
Doctoral degree
Other

Which of the following categories best describes your area of employment (regardless of your actual position)? Check all that apply

Student
Unemployed
Agriculture, forestry, fishing, hunting
Arts, entertainment, recreation
Design/publicity
Education College/University
Education Primary/Secondary
Finance and insurance
Business, marketing, admission
Government and public administration
Health Care, social assistance
Legal services
Scientific or technical services
Software
Transportation
Construction
Manufacturing
Other

Please indicate your total annual household income (in Canadian Dollars)

Less than $20,000
$20,001-$40,000
$40,001-$60,000

$60,001-$80,000
$80,001-$100,000
100,001-$120,000
$120,001-$140,000
$140,001-$160,000
More than $160,000

How many people live in your household including you?

What do you consider your place of residence to be?

Large city or urban area
Suburban area
Small city
Rural area on a farm or ranch
Rural area NOT on a farm or ranch

Thank you for participating in this survey!

Powered by Qualtrics
