# Supplementary material for: Explicit Not Implicit Preferences Predict Conservation Intentions for Endangered Species and Biomes
Source: PLoS One. 2017 Jan 30;12(1):e0170973. doi: 10.1371/journal.pone.0170973 (PMC5279788; doi:10.1371/journal.pone.0170973)
Supplement: S4 Fig — (PDF) [file pone.0170973.s008.pdf]

S4 Fig. Questionnaire for study 3.

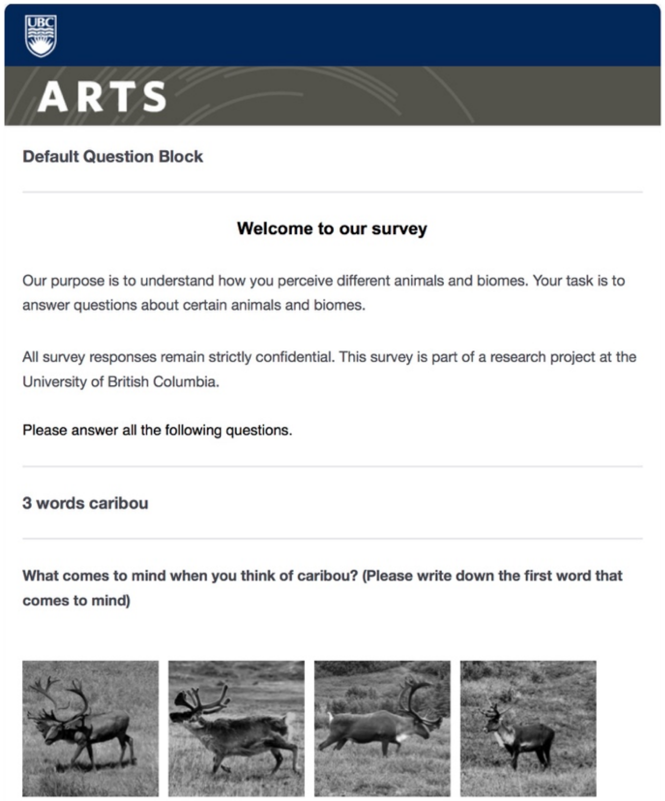
ARTS

Default Question Block

Welcome to our survey

Our purpose is to understand how you perceive different animals and biomes. Your task is to answer questions about certain animals and biomes.

All survey responses remain strictly confidential. This survey is part of a research project at the University of British Columbia.

Please answer all the following questions.

3 words caribou

What comes to mind when you think of caribou? (Please write down the first word that comes to mind)

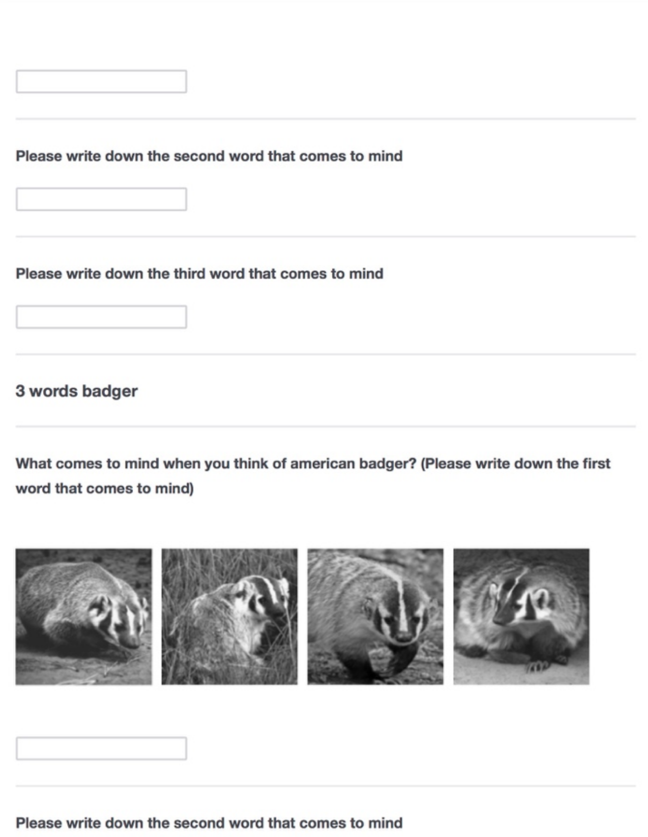
Please write down the second word that comes to mind

Please write down the third word that comes to mind

3 words badger

What comes to mind when you think of american badger? (Please write down the first word that comes to mind)

Please write down the second word that comes to mind

Please write down the third word that comes to mind

3 words sea otter

What comes to mind when you think of sea otter? (Please write down the first word that comes to mind)

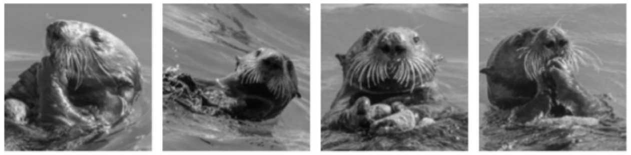

Please write down the second word that comes to mind

Please write down the third word that comes to mind

3 words chat

What comes to mind when you think of yellow-breasted chat? (Please write down the first word that comes to mind)

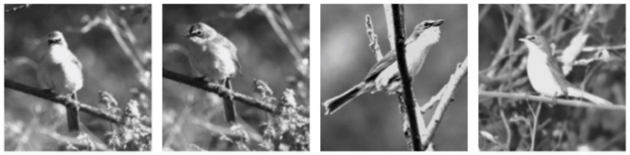

Please write down the second word that comes to mind

Please write down the third word that comes to mind

Donation species

How much money (in US dollars) would you give to conserve each of these animals?

0	American badger
0	Caribou
0	Sea otter
0	Yellow-breasted chat

Like/Familiarity species

Please answer the following questions

	Not at all										Extremely
	0	1	2	3	4	5	6	7	8	9	10
How much do you like **Caribou?**											
How familiar are you with **Caribou?**											

Please answer the following questions

	Not at all										Extremely
	0	1	2	3	4	5	6	7	8	9	10
How much do you like **American badger?**											
How familiar are you with **American badger?**											

Please answer the following questions

	Not at all										Extremely
	0	1	2	3	4	5	6	7	8	9	10
How much do you like **Sea otter?**											
How familiar are you with **Sea otter?**											

Please answer the following questions

	Not at all										Extremely
	0	1	2	3	4	5	6	7	8	9	10
How much do you like **Yellow-breasted chat?**											
How familiar are you with **Yellow-breasted chat?**											

Ranking favorites species

Rank these animals (by dragging and dropping) in order of your most (1) to least (4) favorite

American badger

Caribou

Sea otter

Yellow-breasted chat

Ranking endangered species

Rank these animals (by dragging and dropping) in order of the most (1) to least (4) endangered

American badger

Caribou

Sea otter

Yellow-breasted chat

3 words forest

What comes to mind when you think of forest? (Please write down the first word that comes to mind)

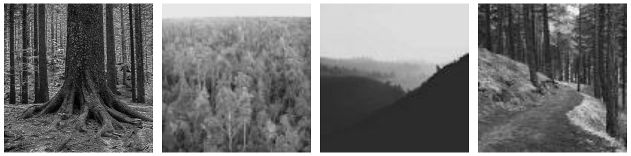

Please write down the second word that comes to mind

Please write down the third word that comes to mind

3 words grassland

What comes to mind when you think of grassland? (Please write down the first word that comes to mind)

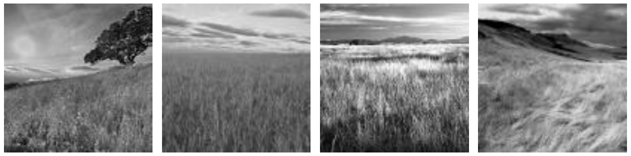

Please write down the second word that comes to mind

Please write down the third word that comes to mind

3 words ocean

What comes to mind when you think of ocean? (Please write down the first word that comes to mind)

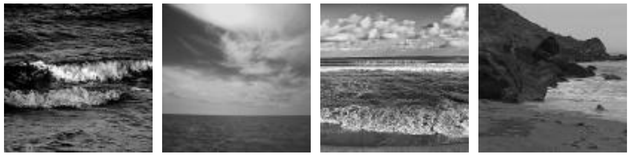

Please write down the second word that comes to mind

Please write down the third word that comes to mind

3 words tundra

What comes to mind when you think of tundra? (Please write down the first word that comes to mind)

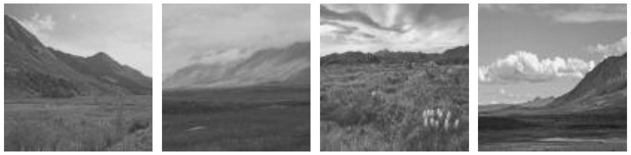

Please write down the second word that comes to mind

Please write down the third word that comes to mind

Donation biomes

How much money (in US dollars) would you give to conserve each of these environments?

- 0 Forest
- 0 Grassland
- 0 Ocean
- 0 Tundra

Like/Familiarity biomes

Please answer the following questions

	Not at all									Extremely
	0	1	2	3	4	5	6	7	8	9	10
How much do you like **forest?**											
How familiar are you with **forest?**											

Please answer the following questions

	Not at all									Extremely
	0	1	2	3	4	5	6	7	8	9	10
How much do you like **grassland?**											
How familiar are you with **grassland?**											

Please answer the following questions

	Not at all									Extremely
	0	1	2	3	4	5	6	7	8	9	10
How much do you like **ocean?**											
How familiar are you with **ocean?**											

Please answer the following questions

	Not at all									Extremely
	0	1	2	3	4	5	6	7	8	9	10
How much do you like **tundra?**											
How familiar are you with **tundra?**											

Ranking favorite biomes

Rank these environments (by dragging and dropping) in order of your most (1) to least (4) favorite

Forest

Grassland

Ocean

Tundra

Ranking threatened biomes

Rank these environments (by dragging and dropping) in order from the most (1) to least (4) threatened

Forest

Grassland

Ocean

Tundra

Conservation behavior

Have you ever been a member of a conservation, animal welfare, or other environmental organization?

Yes
No

Do you have pets?

Yes
No

Have you had pets in the past?

Yes
No

Have you ever gone hunting?

Yes
No

Have you ever gone fishing?

Yes
No

How much time do you spend in nature (public parks, national or provincial parks, forests, etc) on a regular **weekday**?

None
Less than 1/2 an hour
About 1/2 an hour
About 1 hour
2-3 hours
4 or more hours

How much time do you spend in nature (public parks, national or provincial parks, forests, etc) on a regular **weekend day**?

None
Less than 1/2 an hour
About 1/2 an hour
About 1 hour
2-3 hours
4 or more hours

Demographics

In this last section of the survey, we would like to learn more about your background and your current household characteristics. You can be assured that all your answers will be kept confidential. This information will only be used to report results among groups of people. We will never identify individuals or households with these responses.

Age (in years)

Gender

Male
Female
Other

Where do you live? (City or town, Country)

Where are you from? (City or town, Country)

What races or ethnic background do you consider yourself to be? Please check all that apply

White or Caucasian
Black or African-American
Hispanic or Latino (includes Mexican, Central American and South American)
Korean
Japanese
Chinese
Filipino
Pacific islander
Middle eastern
African (NOT African-American)
South Asian (from India, Bangladesh, Pakistan, etc)
Other

What is your religious affiliation?

Mormon
Muslim
an Orthodox Church such as Greek or Russian Orthodox Church
Buddhist
Catholic
Protestant
Jewish
Jehovah's Witness
Hindu
Atheist
Agnostic
Other

What is your political orientation? Rate from a scale from -5= very liberal (left wing) to 5= very conservative (right wing)

	Left wing									Right wing
	-5	-4	-3	-2	-1	0	1	2	3	4	5
Political orientation											

What is the highest level of education you have completed?

High school or equivalent
Vocational/Technical school
College
Bachelor's degree

Professional degree (MD, JD, etc)
Master's degree
Doctoral degree
Other

Which of the following categories best describes your area of employment (regardless of your actual position)? Check all that apply

Student
Unemployed
Agriculture, forestry, fishing, hunting
Arts, entertainment, recreation
Design/publicity
Education College/University
Education Primary/Secondary
Finance and insurance
Business, marketing, administration
Government and public administration
Health Care, social assistance
Legal services
Scientific or technical services
Software
Transportation
Construction
Manufacturing
Other

Please indicate your total annual household income (in US Dollars)

Less than $20.000
$20.001-$40.000
$40.001-$60.000
$60.001-80.000
$80.001-100.000
$100.001-120.000
$120.001-140.000
$140.001-160.000
More than $160.000

How many people live in your household including you?

What do you consider your place of residence to be?

Large city or urban area
Suburban area
Small city or town
Rural area on a farm or ranch
Rural area NOT on a farm or ranch

Thank you for participating in this survey!

Powered by Qualtrics
